# Targeting hypoxic exosomal IGFBP2 overcomes CD47-mediated immune evasion in glioblastoma

**DOI:** 10.1038/s41419-026-08430-9

**Published:** 2026-01-31

**Authors:** Yanhua Qi, Rongrong Zhao, Xinglong Zhang, Huize Xia, Ping Zhang, Qingtong Wang, Shulin Zhao, Shaobo Wang, Hongyu Zhao, Xiaofan Guo, Wei Qiu, Boyan Li, Ziwen Pan, Jiawei Qiu, Zijie Gao, Chengwei Wang, Haiquan Lu, Gang Li, Hao Xue

**Affiliations:** 1 Department of Neurosurgery, Qilu Hospital, Cheeloo College of Medicine and Institute of Brain and Brain-Inspired Science, Shandong University, Jinan, 250012 Shandong P. R. China; 2 Shandong Key Laboratory of Brain Health and Function Remodelling, Jinan, 250012 P. R. China; 3 Department of Neurology, UPMC Stroke Institute, University of Pittsburgh School of Medicine, Pittsburgh, PA USA; 4 Department of Neurosurgery, The Second Hospital, Cheeloo College of Medicine, Shandong University Jinan, 250031 P. R. China; 5 Advanced Medical Research Institute and Key Laboratory for Experimental Teratology of the Ministry of Education, Cheeloo College of Medicine, Shandong University, Jinan, Shandong 250012 China

**Keywords:** Tumour immunology, Tumour immunology

## Abstract

Glioblastoma (GBM) acquires malignant traits through complex molecular adaptations that sustain immune evasion, often characterized by hypoxia and overexpression of the phagocytosis checkpoint CD47. However, the role of hypoxic drivers coordinating CD47-dependent immune evasion remains poorly defined. Here, we integrated single cell RNA sequencing and proteomic analysis to identify that insulin-like growth factor binding protein 2 (IGFBP2) was co-expressed with CD47 in hypoxic mesenchymal-like GBM subpopulations, synergistically promoting tumor progression and immune evasion. Mechanically, hypoxia induced IGFBP2 expression via HIF-2α-mediated transcriptional activation and further increased IGFBP2-positive exosome secretion through HIF-1α-dependent RAB3A upregulation. Moreover, IGFBP2 was predominantly localized on the exosome surface via integrin α5β1 and activated integrin/FAK/STAT3 signaling to enhance CD47 expression and inhibit macrophage phagocytosis. Clinically, serum exosomal IGFBP2 levels correlated with tumor grade and could serve as a diagnostic biomarker. Importantly, combinatorial blockade of IGFBP2 and CD47 synergistically suppressed tumor growth and prolonged survival in orthotopic GBM models. Together, our findings uncovered the hypoxia-exosomal IGFBP2-CD47 axis in GBM immune evasion and provided a compelling rationale for combination therapy to improve immunotherapy efficacy in GBM.

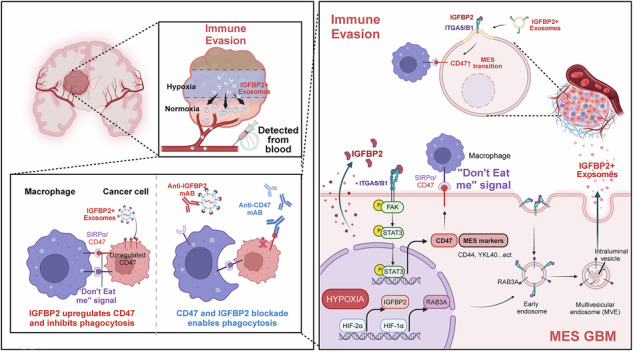

## Introduction

Glioblastoma (GBM) is the most common and malignant tumor of the central nervous system, with a median survival of 15–21 months [1, 2]. The hypoxic microenvironment formed by rapid tumor proliferation drives GBM immune evasion, serving as a critical factor contributing to its high malignancy and therapeutic resistance [3, 4]. Despite the presence of immune cells in hypoxic niches, GBM cells evade phagocytic clearance through multiple mechanisms, including the “don’t eat me” signal induced by CD47-SIRPα interaction [5, 6]. CD47, a transmembrane protein, could transmit a “don’t eat me” signal through its interaction with signal regulatory protein alpha (SIRPα) on macrophages, thereby inhibiting phagocytosis. Immunotherapeutic strategies targeting CD47, such as monoclonal antibodies or small-molecule inhibitors, have demonstrated promising outcomes in preclinical studies in GBM [7]. However, the hypoxic niches in GBM may critically influence the responsiveness to CD47-targeted therapies by upregulating CD47 expression and establishing an immunosuppressive microenvironment [8, 9]. Consequently, these mechanisms significantly undermine the potential of CD47-targeted monotherapy in solid tumors, underscoring the need for combination therapies targeting hypoxia to enhance efficacy.

In the hypoxic microenvironment of GBM, the dynamic interplay between tumor cells and immune cells is a critical driver of immune evasion [10, 11]. Exosomes serve as critical nanoscale mediators of intercellular communication, delivering functional cargos including proteins, coding/non-coding RNAs, and bioactive metabolites to recipient cells [12, 13]. Studies have shown that the release of exosomes is significantly increased under hypoxia, indicating that exosomes play an important role in cell communication within the tumor hypoxic microenvironment [13, 14]. It has been demonstrated that hypoxia induces an increase in CD47 expression in a manner dependent on HIF activation or histone lactylation [9, 15, 16]. In previous studies, we found that hypoxia-induced exosomes could drive the and activation of immunosuppressive cells and upregulate PDL1 expression to promote immune evasion [17, 18]. However, the potential role of hypoxic glioma-derived exosomes in upregulating CD47 expression and their subsequent influence on the effectiveness of CD47-targeted therapies remains to be elucidated. Therefore, targeting the hypoxia-exosome-CD47 axis could provide novel approaches to overcome GBM immune evasion and improve the efficacy of immunotherapy.

In this study, our analysis identified IGFBP2 was highly expressed in both the cells and exosomes under hypoxia, showing a significant positive correlation with CD47. IGFBP2 is a small glycoprotein belonging to the Insulin-like Growth Factor Binding Protein (IGFBP) family. As a secreted protein, IGFBP2 is involved in regulating key cellular processes, including adhesion, migration, and invasion, through its interaction with cell surface receptors such as integrins [19, 20]. Additionally, IGFBP2 plays a critical role in modulating the tumor microenvironment, potentially facilitating immune evasion mechanisms and enhancing the resistance of tumor cells to immune surveillance [21, 22]. Although IGFBP2 is confirmed to be secreted into extracellular space, there are few studies confirming whether IGFBP2 could be secreted into exosome and the exosomal roles of IGFBP2 in glioma are unknown. It has been reported that secreted proteins could bind to the external surface of cancer-derived exosomes via cell surface receptors [23–25]. The advantages of exosome-mediated secreted proteins delivery are summarized as follows: (i) exosomes are equipped with surface proteins, such as integrins and tetraspanins, which allow them to specifically bind to target cells; (ii) exosomes can efficiently cross biological barriers such as the blood-brain barrier (BBB), facilitating the detection of secreted protein in the blood. Therefore, investigating the role of hypoxic exosome-derived IGFBP2 in promoting CD47 expression may enhance the effectiveness of CD47-targeted therapies.

In the present study, we utilized single-cell sequencing to reveal a close association between IGFBP2 and CD47 in the hypoxic niches of GBM. We further confirmed that exosomal IGFBP2 promote the expression of CD47 via integrin/FAK/STAT3 signaling and combined anti-IGFBP2 and anti-CD47 treatment suppressed tumor growth and prolonged the survival time of tumor-bearing mice. Moreover, experiment results showed that the expression of IGFBP2 in exosomes from the serum of GBM patients was significantly higher compared to healthy controls, further supporting the role of exosomal IGFBP2 as a potential diagnostic biomarker.

## Materials & methods

### Cell lines

The mouse GBM cell line GL261 were purchased from the Chinese Academy of Sciences Cell Bank and cultured in DMEM supplemented with 10% FBS. Human GBM stem cells GSC20 and GSC267 were donated by the Bhat lab of MD Anderson Cancer Center and maintained in F12 supplemented with B-27 (Gibco, USA), 20 ng/mL recombinant human (rh) epidermal growth factor (R&D Systems, USA) and 20 ng/mL rh basic fibroblast growth factor (R&D Systems, USA) as previously described. The cell lines were authenticated by short tandem repeat profiling and confirmed to be negative for mycoplasma contamination.

### Patients and specimens

Human glioma tissues, plasma, and normal brain tissues (the cortex of decompressive surgery patients with brain trauma or hypertensive intracerebral hemorrhage) were obtained from patients admitted to Qilu Hospital. All participants provided written informed consent, and the research was approved by the Ethical Committee on Scientific Research of Shandong University Qilu Hospital (approval number: KYLL-2022(ZM)-439).

### Chromatin immunoprecipitation (ChIP) assay

The binding sites of HIF-2α on the promotor region of PDIA3P1 were predicted using JASPAR (http://jaspar.genereg.net/). ChIP assay were performed using the Magna ChIP™ A/G Chromatin Immunoprecipitation Kit (17-10086, Millipore, USA). First, cells are treated with formaldehyde to crosslink proteins to DNA, preserving protein-DNA interactions. The cells are then lysed to release chromatin, which is subsequently sheared into smaller fragments using sonication or enzymatic digestion. The fragmented chromatin is incubated with an antibody specific to the target protein or modification, along with Protein A/G beads, to immunoprecipitate the complexes. After thorough washing to remove non-specific bindings, the crosslinks are reversed by heating, and the DNA is purified using a kit or phenol-chloroform extraction. Finally, the enriched DNA is analyzed by qPCR to identify the DNA regions associated with the target protein. The anti-HIF-2α (Cell Signaling Technology, D6T8V) antibody was used. Co-precipitated DNA was quantified using PCR and RT-qPCR. The IGFBP2 promoter primers used are shown in Supplementary Table 1.

### Tissue dissociation, cDNA synthesis and single cell RNA-Seq library preparation

Collect samples according to the experimental design (PBMCs, Cell lines, tissues). Tissues were washed twice with phosphate buffer saline (PBS). The tissue was cut into small pieces about 1 mm^3^ in size and placed in petri dish with appropriate amount of PBS, then transferred to centrifuge tube, adding appropriate amount of enzyme and shaking at a certain temperature for a period of time; After 2–3 min’ standing, take the supernatant, use a filter membrane to remove large clumps; Following centrifuge the cell and the supernatant was decanted and discarded, resuspended cells with red blood cell lysis buffer and incubation 2–3 min at room temperature and then centrifuge (120 × *g*, 4 °C,3 min), samples were lastly resuspended with PBS. Then, cell suspensions (300–600 living cells per microliter determined by Count Star) were loaded on a Chromium Single Cell Controller (10x Genomics) to generate single-cell gel beads in emulsion (GEMs) by using Single Cell 3’Library and Gel Bead Kit V2 (10x Genomics, 120237) and Chromium Single Cell A Chip Kit (10x Genomics, 120236) according to the manufacturer’s protocol. Single-cell RNA-seq libraries were prepared using Single Cell 3’ Library Gel Bead Kit V2 following the manufacture’s introduction. Finally, sequencing was performed on an Illumina Novaseq6000 with a sequencing depth of at least 100,000 reads per cell and pair end 150 bp (PE150).

### Animal studies

Male C57BL/6 or BALB/c nude mice (aged 4–6 weeks), were obtained from GemPharmatech Co., Ltd. (Nanjing, Jiangsu, China) and housed at the Neurosurgery Laboratory of Qilu Hospital of Shandong University (Jinan, Shandong, China).

To investigate the GBM-promoting effects of IGFBP2 in vivo, GSC267 cells expressing luciferase (5 × 10⁵ per mouse) were implanted intracranially into BALB/c nude mice. Tumor progression was tracked using the IVIS Spectrum in vivo imaging system (IVIS; PerkinElmer Inc., Waltham, MA, USA) on days 4, 7, 14 and 28 post-implantation. Survival analysis was conducted, with survival time defined as the period from tumor implantation to death.

For combination therapy experiments, luciferase-labeled GL261 cells (5 × 10⁵ per mouse) were implanted into the brains of C57BL/6 mice. Treatments included tail vein injections of anti-CD47 reagent (BE0019, BioXcell), anti-IGFBP2 antibody (#AF674, R&D Systems), or isotype control antibody (#BP0089, BioXcell) three times a week after tumor implantation. Bioluminescence imaging was used to assess the tumor volume, using the IVIS Spectrum in vivo imaging system (IVIS; PerkinElmer Inc.). Survival analysis was conducted, with survival time defined as the period from tumor implantation to death. Brains were collected and fixed in 4% formaldehyde for HE staining and IHC analysis. The euthanasia procedures were performed in accordance with Institutional Animal Care and Use Committee (IACUC) guidelines and were approved by the Animal Care and Use Committee of the Qilu Hospital of Shandong University (Approval No. DWLL-2023-169).

### Statistical analysis

Statistical analyses were performed with GraphPad Software 8 (GraphPad, CA, USA) using R Studio (version 4.3.1). Kaplan-Meier curves were analyzed using log-rank tests, and Cox regression models (α = 0.05) were applied via the coxph function in the R survival package, using the Breslow method for tied events. Correlations were assessed with Pearson tests. Data are shown as mean ± SD. Student’s *t* test, Wilcoxon test, or one-way ANOVA were used for group comparisons as appropriate. *P* < 0.05 was considered significant.

## Results

### IGFBP2 is positively correlated with CD47 in MES GBM of hypoxic niche

**Fig. 1:**
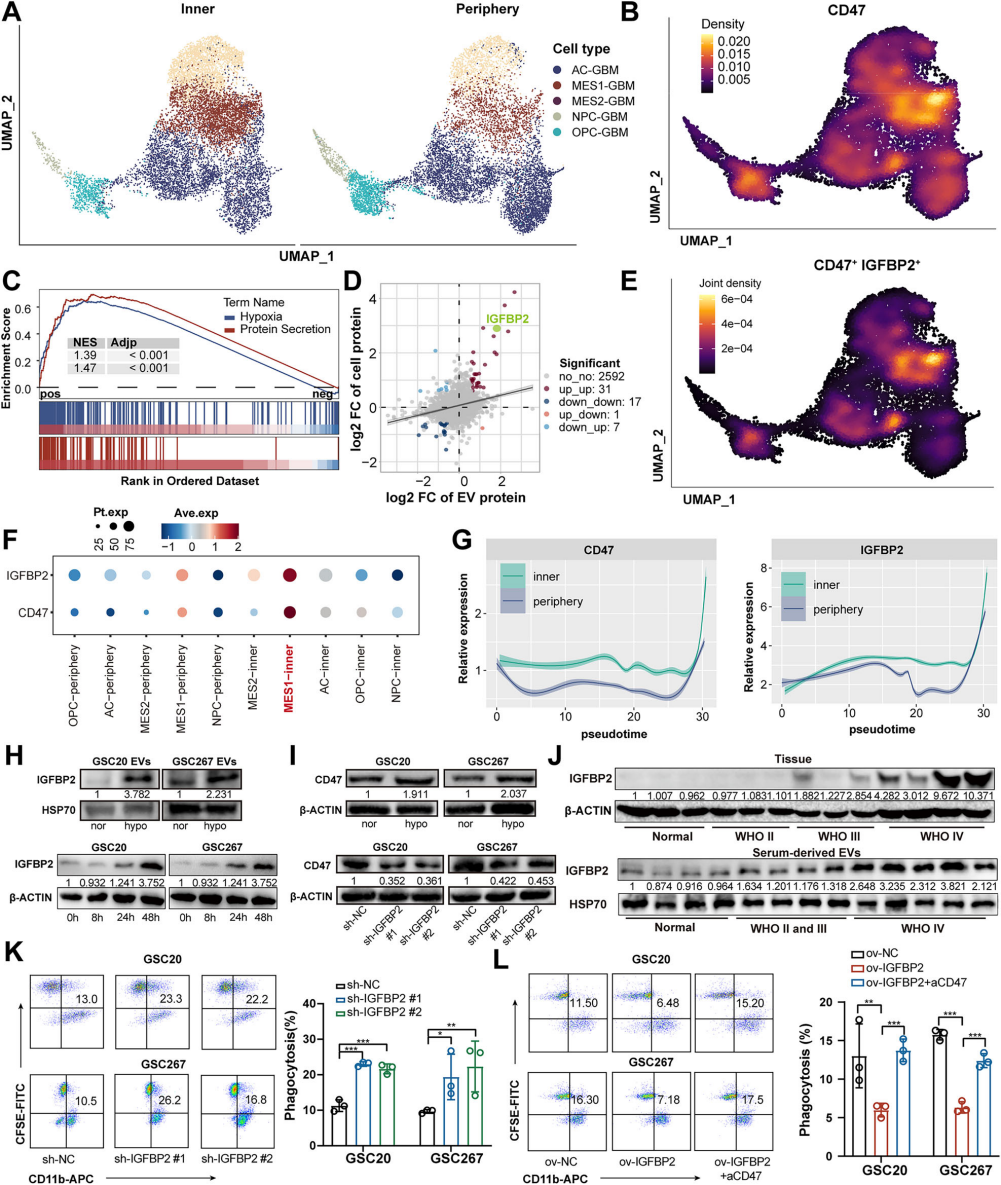
IGFBP2 is positively correlated with CD47 in hypoxic niches of GBM. **A** Umap and density plots showing MES-like GBM subpopulation was more enriched in tumor core. Tissues at two distinct locations (Inner: tumor core; Periphery: tumor invasive margin) were collected. **B** Umap and density plots showing CD47 was highly expressed in the MES-like GBM subpopulation. **C** GSEA of scRNA data indicating that positive correlated genes with CD47 was significantly enriched in Hypoxia and Protein secretion. **D** Proteomic sequencing results confirm a significant upregulation of IGFBP2 in both GBM cells and exosomes under hypoxic conditions. **E** Umap and density plots showing IGFBP2 and CD47 were co-expressed in the MES1-like GBM subpopulation. **F** Bubble plot indicating that CD47 and IGFBP2 were highly expressed in the MES1-inner subpopulation. **G** Monocle2 pseudotime analysis revealing that the expression of CD47 and IGFBP2 was gradually upregulated with tumor progression. **H** Western blot assays showing IGFBP2 was upregulated in GBM cells and exosomes under hypoxia. **I**.Western blot assays showing CD47 was upregulated in GBM cells under hypoxia and decreased after IGFBP2 knockdown. **J** Western blot assays confirmed a significant increase in IGFBP2 expression in both tumor tissues and plasma exosomes of high-grade glioma patients. **K** Flow cytometry analysis and quantification of macrophages phagocytosis in GSC20 and GSC267 cells transfected with sh-NC or sh-IGFBP2. Right graph: Quantification of the percentage of macrophages phagocytosis. (*n* = 3) **L** Flow cytometry analysis and quantification of macrophages phagocytosis in GSC20 cells transfected with ov-NC or ov-IGFBP2 and treated with IgG or blocking antibody anti-integrin α5β1. Right graph: Quantification of the percentage of macrophages phagocytosis. (*n* = 3) Data are presented as the mean ± SD. Statistical significance was determined using one-way ANOVA (*P < 0.05; **P < 0.01; ***P < 0.001).

To investigate the relationship between the hypoxic microenvironment of GBM and immune evasion, we collected GBM tissues from GBM patients at distinct locations (Inner: tumor core; Periphery: tumor invasive margin) and performed single-cell RNA-seq (scRNA) with the 10×Genomics Chromium System (Fig. S1A). In accordance with previous annotations [26], tumor cells were classified into four major malignant subtypes, including neural progenitor-like GBM cells (NPC-GBM), oligodendrocyte progenitor-like GBM cells (OPC-GBM), astrocytic-like GBM cells (AC-GBM), as well as mesenchymal-like GBM cells (MES-GBM), which was further divide into MES1-GBM and MES2-GBM subpopulations (Fig. 1A, Fig. S1B). And we found that MES-GBM demonstrated a higher distribution proportion within the tumor core compared to the peripheral regions (Fig. 1A, Fig. S1C). Further GSVA functional enrichment analysis revealed that MES1-GBM subpopulation exhibited highly complex functional characteristics, concomitant with aberrant activation of multiple oncogenic signaling pathways, including Epithelial Mesenchymal Transition (EMT), Angiogenesis, and hypoxic activity, which were notably elevated in the tumor core (inner region) compared to peripheral areas (Fig. S1D). Our integrated scRNA analysis delineated a hypoxic-driven immunosuppressive niche within the GBM core, where the MES1-GBM subpopulation orchestrated oncogenic pathway activation.

Next, we explored the regulatory landscape of CD47 in GBM cells, we identified that CD47 was highly expressed in the MES1-GBM subpopulation (Fig. 1B), and GSEA revealed that genes positively associated with CD47 were significantly enriched in hypoxia and protein secretion-related pathways (Fig. 1C, Fig. S2A), which were robustly activated in MES1-GBM subpopulations and notably elevated in the tumor inner region (Fig. S1D). To further investigate the effect of hypoxia-regulated proteins on CD47 in GBM cells, we then isolated the secreted exosomes of hypoxia-stimulated GSCs, which exhibited similar typical cup-shaped morphologies, sizes, and concentrations (Fig. S2B–D), and exosome uptake experiments confirmed that glioma-derived exosomes can be internalized by tumor cells (Fig. S2E). Moreover, we performed proteomic sequencing to analyze the protein compositions of both GBM cells and exosomes under hypoxic conditions and demonstrated that IGFBP2, which positively correlated with CD47 (Fig. S2A), was markedly upregulated in both hypoxic GBM cells and exosomes compared to normoxia (Fig. 1D). Further scRNA density analyses demonstrated that IGFBP2 and CD47 exhibited specific co-expression within the MES1-GBM subpopulation (Fig. 1E), with marked enrichment localized specifically to the inner MES1-GBM cells (Fig. 1F). Furthermore, monocle2-based pseudotemporal trajectory analysis revealed a progressive upregulation of CD47 and IGFBP2 expression during GBM progression, with higher expression in the inner hypoxia region (Fig. 1G, Fig. S2F). Immunofluorescence analysis of GBM tissues confirmed that IGFBP2-positive exosomes accumulate in hypoxic regions, where IGFBP2 and CD47 are colocalized (Fig. S2G, H). Western blot and flow cytometry experiments further confirmed that expression of CD47 and IGFBP2 were upregulated under hypoxic conditions and CD47 was decreased after the knockdown of IGFBP2 and treated with anti-IGFBP2 antibody (Fig. 1H, I, Fig. S2I–K). Moreover, our results showed that IGFBP2-knockdown cells were more susceptible to phagocytosis by macrophages than the control cells (Fig. 1K). Consistently, overexpression of IGFBP2 were more likely to escape phagocytosis by macrophages, which could be rescued via neutralizing CD47 antibody (aCD47) (Fig. 1L).

We then collected tumor tissues of GBM patients and isolated exosomes from the plasma samples of patients with GBM and healthy donors. Western blot and immunohistochemical analyses demonstrated that IGFBP2 was highly expressed in both tumor tissues and serum-derived exosomes from GBM patients, with its expression levels increased with the progression of tumor grade (Fig. 1J, Fig. S2L), suggesting that IGFBP2 had potential as a biomarker for glioma diagnosis and pathological grading. Collectively, we proposed that hypoxia-driven co-expression of CD47 and IGFBP2 in MES1-GBM subpopulations promoted tumor progression and exosome-mediated signaling, with IGFBP2 serving as a hypoxia-inducible diagnostic biomarker correlated to CD47-mediated immune escape.

### IGFBP2 is upregulated by HIF-2α under hypoxia

**Fig. 2:**
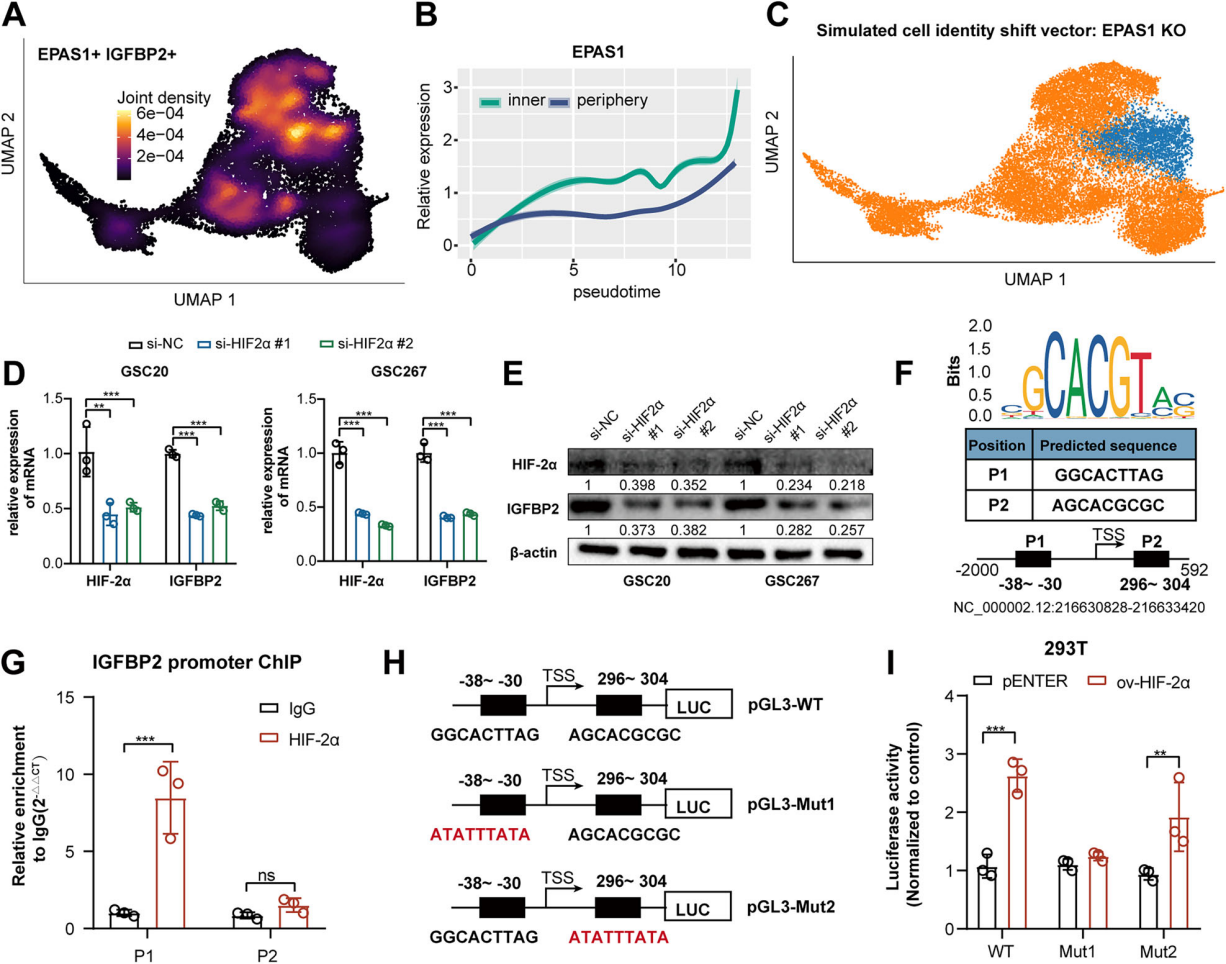
IGFBP2 is upregulated by HIF-2α under hypoxia. **A** Umap and density plots showing that IGFBP2 and EPAS1 were co-expressed in the MES1-like GBM subpopulation. **B** Monocle2 pseudotime analysis revealing that the expression of HIF-2α was gradually upregulated with tumor progression. **C** Umap and density plots showing that EPAS1 knockout perturbation suppresses the transition trajectory of the MES1-GBM subpopulation state. **D**, **E** qPCR assay and western blot assay showing the expression of HIF2α and IGFBP2 treated with si-NC or si-HIF2α. **F**, **G** ChIP-qPCR analysis indicated higher fold enrichment of promoter site P1 in the anti-HIF-2α antibody group compared to the IgG group in GSC267 cells. **H**, **I** Dual luciferase assay showed HIF2α overexpression could increase promoter activity in HEK-293T cells transfected with plasmid WT and Mut2 and no increase occurred in Mut1 transfected cells. Data are presented as the mean ± SD. Statistical significance was determined using one-way ANOVA and (*P < 0.05; **P < 0.01; ***P < 0.001).

Given that IGFBP2 is specifically overexpressed in the MES1-GBM subpopulation, we further investigated the transcriptional regulatory mechanisms driving its specific overexpression. Intersection analysis of the top 30 genes positively correlated with IGFBP2 (Fig. S3A) and the top 10 transcription factors specifically enriched in the MES1-GBM subpopulation (Fig. S3B) identified EPAS1, whose protein name is HIF-2α, as a key regulator co-expressed with IGFBP2 within this population (Fig. 2A). Furthermore, monocle2 pseudotime analysis revealed that the expression of EPAS1 gradually increased with tumor progression, with the expression was higher in inner regions (Fig. 2B). We then performed EPAS1 knockout using CellOracle [27], and found that EPAS1 knockout perturbation suppresses the transition trajectory of the MES1-GBM subpopulation state (Fig. 2C). Further RT-qPCR and western blot assays revealed that HIF-2α knockdown, rather than HIF-1α knockdown, blocked the expression of IGFBP2 mRNA and protein in cells exposed to 1% O2 (Fig. 2D, E, Fig. S3C-D).

**Fig. 3:**
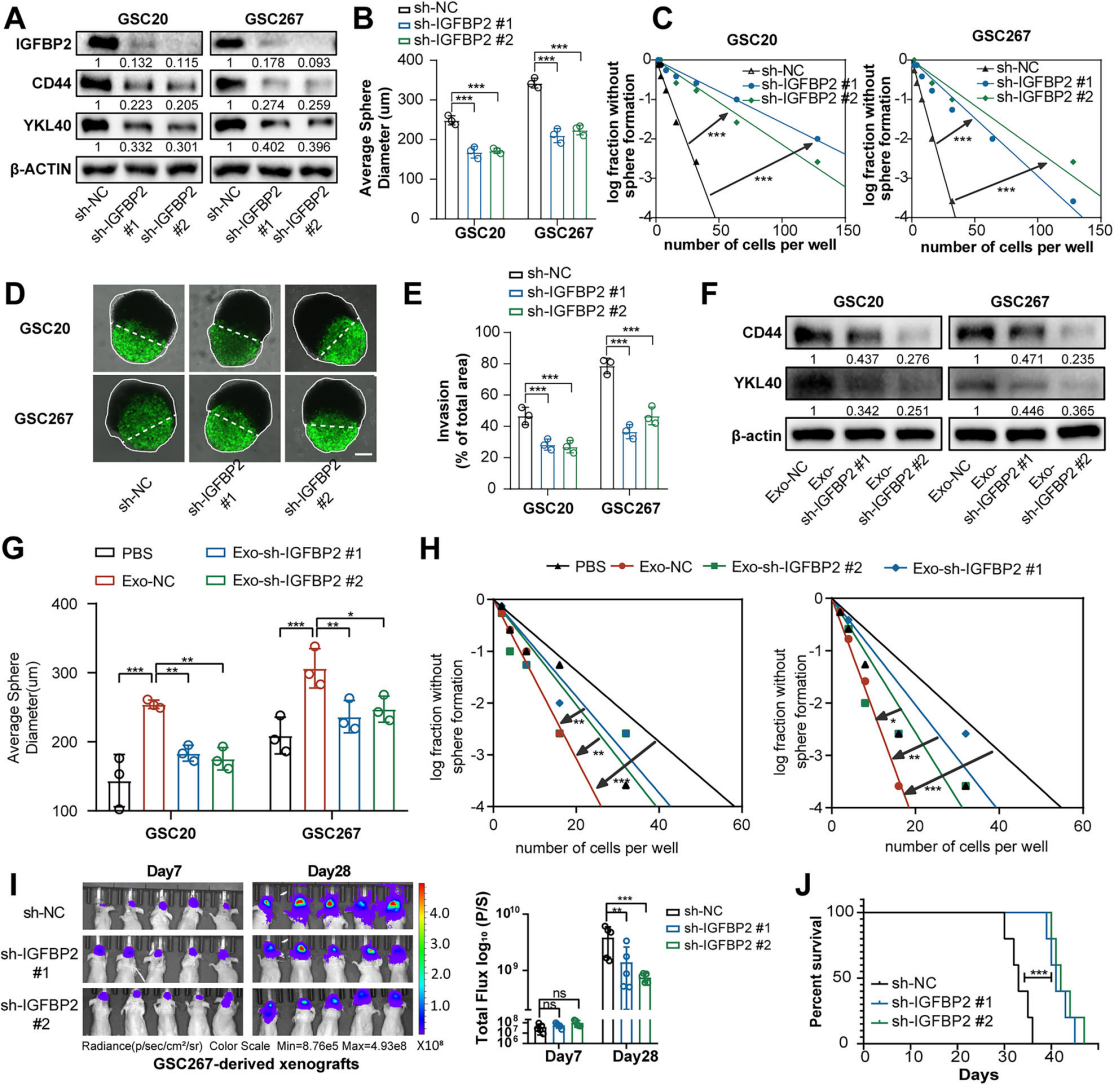
IGFBP2 promotes MES transformation and tumorigenesis in GBM. **A** Western blot assays showing the expression change of MES phenotype markers (CD44 and YKL40) and IGFBP2 in GSC20 and GSC267 cells transfected with sh-NC or sh-IGFBP2. **B** Quantified analysis of spheres diameter of tumor spheres formation after GSC20 and GSC267 cells transfected with sh-NC or sh-IGFBP2. **C** Limiting dilution assay for GSC20 and GSC267 cells transfected with sh-NC or sh-IGFBP2. **D**, **E** Ex vivo co-culture invasion assays for GSC20 and GSC267 cells transfected with sh-NC or sh-IGFBP2. (*n* = 3). The invasion ability was evaluated at 72 h. Scale bar = 200 μm. **F** Western blot assays showing the expression change of MES phenotype markers (CD44 and YKL40) in GSC20 and GSC267 cells treated with exosome derived from the cell supernatant. **G** Quantified analysis of spheres diameter of tumor spheres formation after GSC20 and GSC267 cells treated with exosome derived from the cell supernatant. **H** Limiting dilution assay for GSC20 and GSC267 cells treated with exosome derived from the cell supernatant. **I** Bioluminescence imaging of xenograft models established with GSC267 cells transfected with sh-NC or sh-IGFBP2 on the indicated days after surgery. In vivo tumor activities were assessed by bioluminescent in vivo imaging system on the indicated days after surgery. *n* = 5. **J** Kaplan-Meier survival curves for animals in different groups, *n* = 5 for each group. Data are presented as the mean ± SD. Statistical significance was determined using one-way ANOVA and the log-rank test (*P < 0.05; **P < 0.01; ***P < 0.001).

To determine whether HIF-2α directly binds to the IGFBP2 gene promoter and activates its transcription in GSC cells under hypoxia, the JASPAR database was utilized to predict two HIF-2α binding sites (P1, P2) on the IGFBP2 promoter region (Fig. 3F). Chromatin immunoprecipitation (ChIP), followed by qPCR, confirmed HIF-2α’s binding to P1 site on the IGFBP2 promoter (Fig. 2G). The Dual-Luciferase Reporter Assay validated that overexpression of HIF-2α substantially increased the promoter activity of IGFBP2, while mutations in the HIF-2α binding region P1 of the IGFBP2 reporter abrogated the effect of HIF-2α (Fig. 2H, I). Taken together, these data demonstrated that IGFBP2 expression was induced under hypoxia in a HIF-2α dependent manner.

### IGFBP2 promotes MES transformation and tumorigenesis in GBM

Next, we investigated the relationship between IGFBP2 and the MES transition of GBM cells. ScRNA GSEA analysis showed that genes positively associated with IGFBP2 were significantly enriched in MES1 and EMT enrichment (Fig. S4A). And IGFBP2 was higher expressed in MES-subtype GSCs, compaered to PN-subtype (Fig. S4B). And western blot analysis demonstrated that knockdown of IGFBP2 suppressed the expression of MES-GBM markers (CD44 and YKL40) (Fig. 3A). Furthermore, we performed neurosphere formation and limiting dilution assays on MES GSCs (20 and 267), demonstrating that IGFBP2 significantly promoted neurosphere expansion and the sphere formation ability of GSCs (Fig. 3B-C, Fig. S4C). MES-GBM exhibits highly invasive characteristics [28], prompting us to establish an invasion model of GBM tumor spheroids co-cultured with normal rat brain organoids, as previously described [29, 30], to more accurately mimic the physiologically invasive tumor microenvironment. In this ex vivo model, GBM tumor spheroids with IGFBP2 knockdown were less invasive into rat brain tissue than control GBM tumor spheroids (Fig. 3D, E). Additionally, we treated GSCs with sh-NC or sh-IGFBP2 exosomes, and the results showed that exosomes with IGFBP2 knockdown exhibit a diminished capacity to promote the MES transition in GBM (Fig. 3G, H, Fig. S4D-E).

To evaluate the effect of IGFBP2 in vivo, GSC267 were implanted into mouse brains (4-week-old nude mice) to generate orthotopic xenografts. It revealed that knockdown of IGFBP2 significantly inhibited tumor growth and prolonged the survival time of tumor-bearing mice (Fig. 3I, J, Fig. S4F-G). Taken together, these data demonstrate that IGFBP2 significantly promoted MES transformation and tumorigenesis of GBM via cell-autonomous and exosome-dependent intercellular signaling.

### Hypoxia enhances exosomal surface localization of IGFBP2 via integrin α5β1-mediated binding

**Fig. 4:**
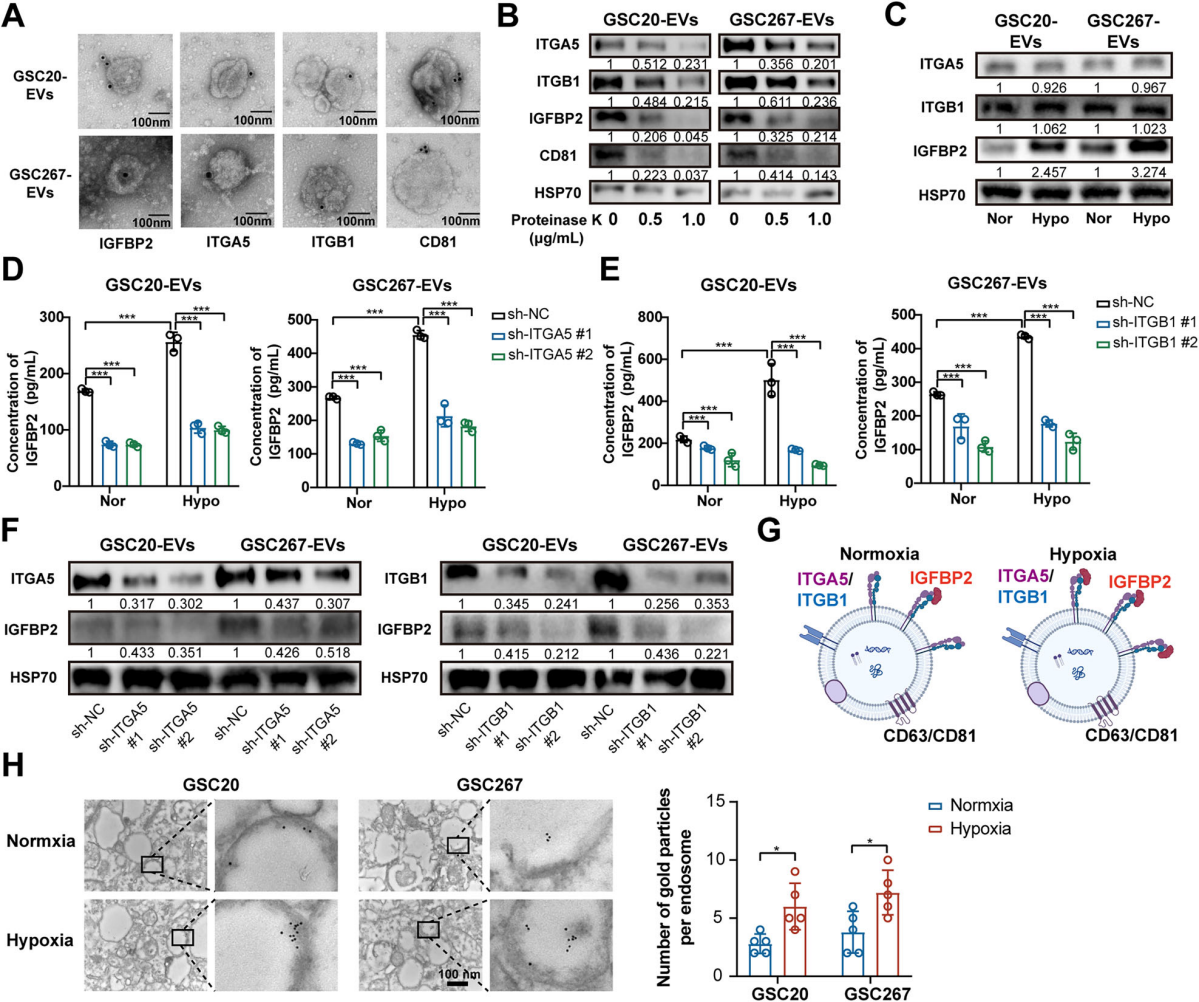
IGFBP2 is localized on the surface of exosomes by binding to integrin α5β1. **A** Representative electron micrographs of exosomes derived from the cell supernatant of GSC20 and GSC267 cells. Immunogold labeling of exosomes using anti-IGFBP2, anti-ITGA5, anti-ITGB1 and anti-CD81 antibodies followed by secondary antibodies conjugated to 10 nm gold particles. Scale bar = 100 nm. **B** Western blot assays showing the protein expression of IGFBP2, ITGA5, ITGB1, CD81 and HSP70 in exosomes derived from the cell supernatant of GSC20 and GSC267 cells treated with increasing concentrations of proteinase. **C** Western blot assays showing the protein expression of IGFBP2, ITGA5, ITGB1 and HSP70 in exosomes derived from the cell supernatant of GSC20 and GSC267 cells under normoxic and hypoxic condition. **D**, **E** Quantification of IGFBP2 on exosomes derived from the cell supernatant of GSC20 and GSC267 cells transfected with sh-NC, sh-ITGA5 or sh-ITGB1 by ELISA. **F** Western blot assays showing the protein expression of IGFBP2, ITGA5, ITGB1 and HSP70 in exosomes derived from the cell supernatant of GSC20 and GSC267 cells transfected with sh-NC, sh-ITGA5 or sh-ITGB1. **G** A schematic diagram illustrating IGFBP2 localized on the surface of exosomes by binding to integrin α5β1 under normoxia and hypoxia. **H** Representative electron micrographs of the localization of IGFBP2 in GSC20 and GSC267 cells under normoxia and hypoxia. Immunogold labeling using anti-IGFBP2 antibodies followed by secondary antibodies conjugated to 10 nm gold particles. Scale bar = 100 nm. Right graph: Quantification of the number of IGFBP2^+^ particles per cell. The dot plot represents the number of IGFBP2^+^ particles from individual cells (*n* = 5). Data are presented as the mean ± SD. Statistical significance was determined using one-way ANOVA and (*P < 0.05; **P < 0.01; ***P < 0.001).

Recent study report that secreted proteins in the tumor microenvironment could bind to the surface of exosomes secreted by cancer cells [23, 25]. We next examined whether IGFBP2 was localized within exosomes or present on the surface of exosomes. First, our immunogold labeling indicated that IGFBP2 and integrin α5β1 were present on the membrane of exosomes (Fig. 4A). Then, we treated GBM-derived exosomes with proteinase K to degrade proteins that led to the degradation of the outer membrane proteins such as CD81, but not the intravesicular HSP70. The findings showed that IGFBP2 and integrin α5β1 were localized on the surface of exosomes surface and hypoxia upregulates the levels of IGFBP2 in exosomes (Fig. 4B, C).

To further confirm that integrin α5β1 mediate the localization of IGFBP2 on the exosomal membrane surface, we knocked down integrin α5β1 in GSC cells and the level of IGFBP2 was greatly reduced compared to the control group (Fig. 4D–F). Therefore, we proposed that IGFBP2 is localized on the exosomal surface through its interaction with integrin α5β1, with an increased binding of IGFBP2 to the exosomal membrane under hypoxic conditions (Fig. 4G). Since the endosomal system is crucial for the formation of multivesicular bodies (MVBs) and the secretion of MVB-derived exosomes [12, 31], we conducted an immunoelectron microscopy experiment and found that IGFBP2 was more abundantly enriched in endosomes under hypoxia (Fig. 4H), which further confirmed that before secretion on the exosomes, a higher amount of IGFBP2 was endocytosed into the cells under hypoxic conditions. Overall, we confirmed that hypoxia enhanced IGFBP2 exosomal surface localization via integrin α5β1-mediated binding.

### Exosomal IGFBP2 promotes the CD47-mediated immune evasion via integrin/FAK/STAT3 signaling

**Fig. 5:**
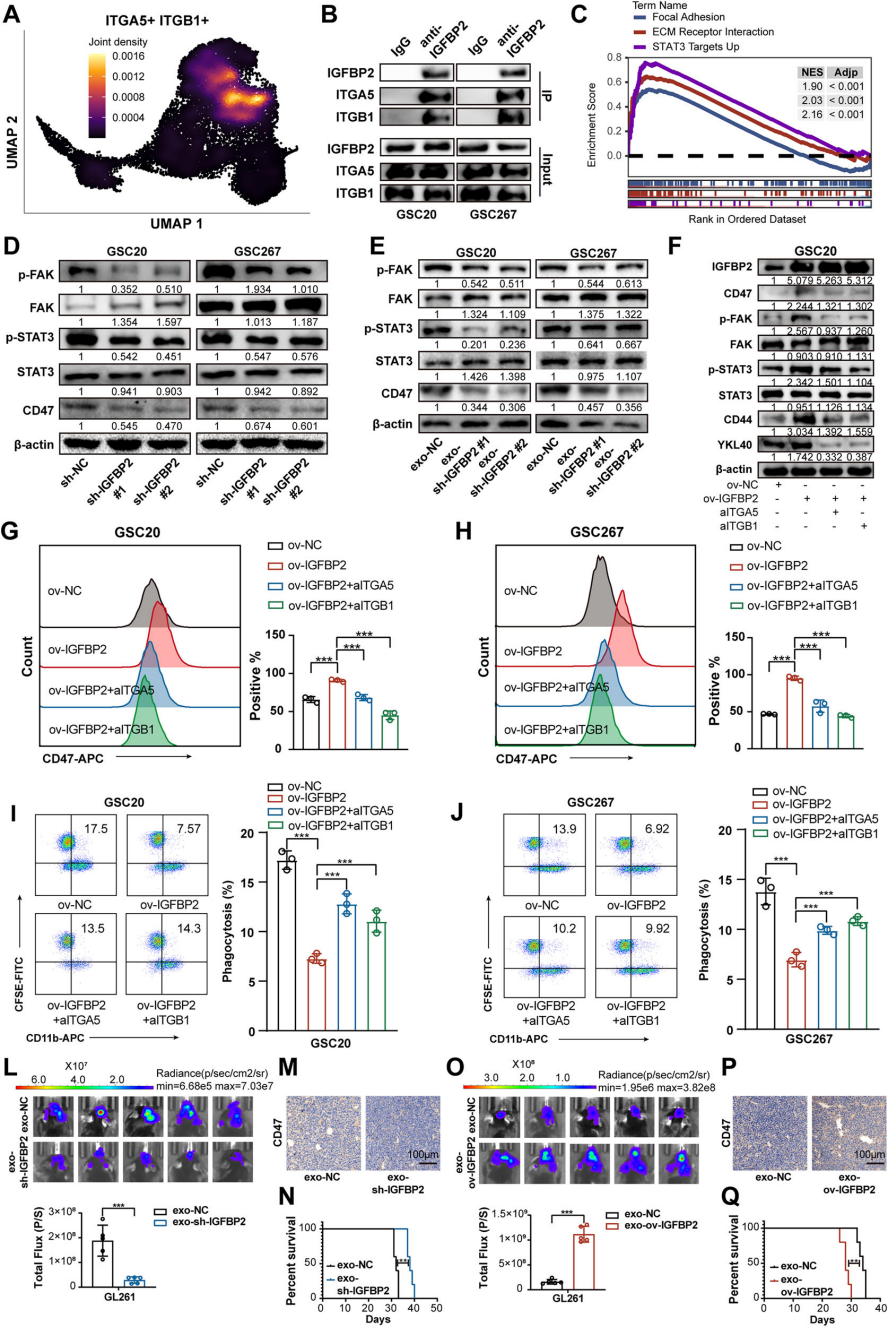
Exosomal IGFBP2 interacts with integrin α5β1 to upregulate CD47 expression via FAK-STAT3 signaling. **A** Umap and density plots showing specific co-expression of IGFBP2, ITGA5 and ITGB1 within the MES1-GBM subpopulation. **B** Co-IP and western blot assays showing the interaction of IGFBP2, ITGA5 and ITGB1 using anti-IGFBP2 antibody. **C** GSEA of scRNA data indicating that positive correlated genes with IGFBP2 was significantly enriched in “focal adhesion”, “extracellular matrix (ECM) receptor interaction” and “STAT3 signaling pathway” pathways. **D** Western blot assays showing the protein expression of FAK-STAT3 signaling pathway and CD47 in GSC20 and GSC267 cells transfected with sh-NC or sh-IGFBP2. **E** Western blot assays showing the protein expression of FAK-STAT3 signaling pathway and CD47 in GSC20 and GSC267 cells treated with exosome derived from the cell supernatant. **F** Western blot assays showing the protein expression of FAK-STAT3 signaling pathway, MES phenotype markers (CD44 and YKL40) and CD47 in GSC267 cells with transfected with ov-NC or ov-IGFBP2 and treated with IgG or blocking antibody anti-integrin α5β1. **G**, **H** Flow cytometry analysis of CD47 expression on GSC20 and GSC267 cells transfected with ov-NC or ov-IGFBP2 and treated with IgG or blocking antibody anti-integrin α5β1. Right graph: Quantification of the percentage of CD47^+^ cells. (*n* = 3) **I**, **J** Flow cytometry analysis and quantification of macrophages phagocytosis in GSC20 and GSC267 cells transfected with ov-NC or ov-IGFBP2 and treated with IgG or blocking antibody anti-integrin α5β1. Right graph: Quantification of the percentage of macrophages phagocytosis. (*n* = 3). **L** Bioluminescence imaging of xenograft models established with GSC267 cells treated with sh-NC-exosome or sh-IGFBP2-exosome. In vivo tumor activities were assessed by bioluminescent in vivo imaging system. *n* = 5. **M** Representative IHC-staining images showing CD47 expression of tumor tissues in different groups. Scale bar, 100 μm. **N** Kaplan-Meier survival curves for animals in different groups, *n* = 5 for each group. **O** Bioluminescence imaging of xenograft models established with GSC267 cells treated with ov-NC-exosome or ov-IGFBP2-exosome. In vivo tumor activities were assessed by bioluminescent in vivo imaging system. *n* = 5. **P** Representative IHC-staining images showing CD47 expression of tumor tissues in different groups. Scale bar, 100 μm. **Q** Kaplan-Meier survival curves for animals in different groups, *n* = 5 for each group. Data are presented as the mean ± SD. Statistical significance was determined using one-way ANOVA (*P < 0.05; **P < 0.01; ***P < 0.001).

Next, we investigated the mechanism underlying the regulation of CD47 expression by IGFBP2. It has been reported that IGFBP2 interacts with integrin receptors to facilitate the malignant progression in various tumors [32, 33]. Our Co-IP assay and mass spectrometry sequencing data indicated that integrin α5β1 (ITGA5/ITGB1) were all potential proteins bind to IGFBP2 (Fig. S5A, Table S4). And scRNA analyses also demonstrated that ITGB1 and ITGA5 exhibited specific co-expression within the MES1-GBM subpopulation (Fig. 5A, Fig. S5C-D), with marked enrichment localized specifically to the inner MES1-GBM cells (Fig. S5C). Our subsequent Co-IP assays demonstrated that IGFBP2 could bind integrin α5β1 (ITGA5/ITGB1) in GSC20 and GSC267 (Fig. 5B, Fig. S5B). Intergrins could activate the FAK to regulate several downstream signaling pathways including signal transducer and activator of transcription 3 (STAT3) [34], which could upregulate the expression of CD47 [15, 35]. scRNA-seq GSEA revealed that genes positively correlated with IGFBP2 exhibited significant enrichment in “focal adhesion”, “extracellular matrix (ECM) receptor interaction” and “STAT3 signaling pathway” pathways (Fig. 5C), suggesting potential mechanistic crosstalk between IGFBP2 and the FAK/STAT3 signaling. Further experiments confirmed that knockdown of IGFBP2 could inhibit the FAK-STAT3 signaling pathway and downregulate CD47 expression in GBM via cell-autonomous and exosome-dependent intercellular signaling (Fig. 5D, E). Moreover, overexpression of IGFBP2 could activate the FAK-STAT3 signaling pathway and upregulate CD47 expression, and this effect can be rescued by treatment with specific STAT3 inhibitors (Stattic, 5 μM) and FAK inhibitors (Y15, 10 μM) (Fig. S5E-F). Consistently, overexpression of IGFBP2 were more likely to escape phagocytosis by macrophages by activating FAK-STAT3 pathway in GSCs, which could be rescued via neutralizing ITGB1 antibody (aITGB1) and neutralizing ITGA5 antibody (aITGA5) (Fig. 5F–J, Fig. S5G).

We further investigated the effects of exosomes derived from IGFBP2-knockdown or IGFBP2-overexpressing cancer cells on CD47 expression and macrophage phagocytosis. Western blot analysis confirmed that exosomes derived from IGFBP2-knockdown cells reduced CD47 expression, whereas exosomes derived from IGFBP2-overexpressing cells enhanced CD47 expression (Fig. 5E, Fig. S5J). In vitro assays demonstrated that treatment with IGFBP2-deficient exosomes weakened the inhibitory effect on macrophage phagocytosis, while IGFBP2-overexpressing exosomes markedly suppressed macrophage phagocytic activity (Fig. S5H-I). Consistently, in vivo experiments showed that intravenous injection of IGFBP2-knockdown exosomes into mice slowed tumor growth and increased macrophage phagocytosis compared with controls, whereas IGFBP2-overexpressing exosomes had the opposite effect (Fig. 5L–Q, Fig. S5K-L). Collectively, these data demonstrated that exosomal IGFBP2 promoted CD47-mediated immune evasion in GBM by engaging integrin α5β1 to activate the FAK-STAT3 signaling axis.

### Hypoxia induces the secretion of IGFBP2-positive exosomes by upregulating RAB3A

**Fig. 6:**
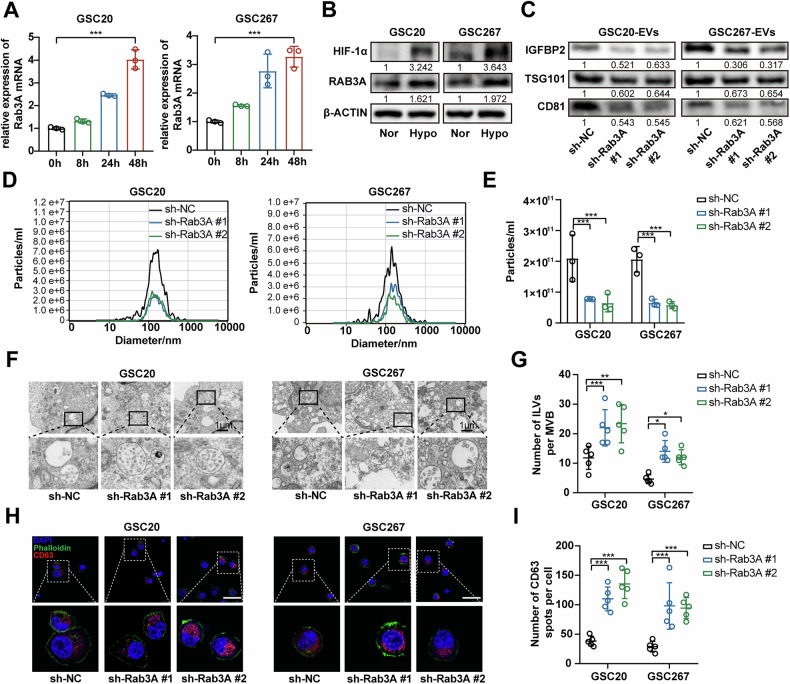
RAB3A is upregulated to increase the secretion of exosomes under hypoxia. **A**, **B** qPCR assay and western blot assay showing the expression of RAB3A under hypoxia. **C** Western blot assays showing the protein expression of HIF-1α, RAB3A, β-actin in GSC20 and GSC267 cells under hypoxic condition. **D**, **E** The size and quantification of exosomes derived from the cell supernatant of GSC20 and GSC267 cells transfected with sh-NC or sh-RAB3A were measured using NTA. Exosomes were collected from 20 × 10^6^ cells for each group. **F**, **G** Representative electron micrographs of MVBs in GSC20 and GSC267 cells transfected with sh-NC or sh-Rab3A. Right graph: quantification of ILV number per MVB. Each dot represents the number of ILVs per MVB (*n* = 5 per group). Scale bar = 1 μm. **H**, **I** Confocal microscopic analysis of Rab3A-KD and Ctrl-KD cells stained with anti-CD63. Scale bar = 50 μm. Right graph: Quantification of the number of CD63^+^ particles per cell. The dot plot represents the number of CD63^+^ particles from individual cells (*n* = 5). Data are presented as the mean ± SD. Statistical significance was determined using one-way ANOVA and (*P < 0.05; **P < 0.01; ***P < 0.001).

It has been reported that hypoxia increases the release of exosomes in various tumor and non-tumor cells [36, 37]. We hypothesize that, under hypoxic conditions, GBM cells secrete more IGFBP2-positive exosomes into the tumor microenvironment, thereby enhancing CD47 expression throughout the entire tumor microenvironment. However, the detailed mechanism by which hypoxia increases exosome release from GBM cells is still not well understood. We analyzed RNA sequencing of GSE45117 and GSE232725 and found RAB3A was upregulated in GBM cells under hypoxia (Fig. S6A), identified as a critical regulator in exosome biogenesis, particularly in modulating exosome secretion and vesicle formation [38]. Rab GTPases, including the RAB3A/RAB27A system, represent a crucial family of proteins implicated in vesicles secretion [39, 40]. RT-qPCR and western blot assays confirmed that RAB3A was increased under hypoxia (Fig. 6A, B) and knockdown of HIF-1α suppressed the expression of RAB3A in GSC cells (Fig. S6B-C).

To evaluate the effects of RAB3A on exosomes secretion in GBM cells, we knocked down RAB3A to detect number of exosomes secreted from GSC cells (Fig. S6D). The measurement of exosomal IGFBP2 protein levels and nanoparticle tracking analysis (NTA) showed a decrease in exosomes secretion after RAB3A knockdown under hypoxia (Fig. 6C–E). To further investigate the mechanisms by which RAB3A knockdown reduces exosome secretion, we conducted electron microscopy to examine the number and morphology of Intraluminal vesicles (ILVs) and MVBs. Interestingly, while exosome secretion significantly decreased with Rab3A depletion, the number of ILVs per MVB increased dramatically compared to control cells (Fig. 6F, G). Consistently, we performed an immunofluorescence assay using CD63 as a marker of MVBs and found that the number of MVBs increased due to impaired exosome secretion (Fig. 6H, I). This suggests that the absence of RAB3A may influence exosome release and lead to an abnormal accumulation of ILVs within MVBs. Therefore, these data revealed that hypoxia increased IGFBP2 expression through HIF-2α-dependent transcriptional activation and further enhanced the secretion of IGFBP2-positive exosomes via HIF-1α-upregulated RAB3A. These findings elucidate the mechanism of hypoxia-driven immune evasion in GBM through enhanced release of IGFBP2-positive exosomes.

### Combinatorial blockade of IGFBP2 and CD47 synergistically suppresses GBM malignant pression

**Fig. 7:**
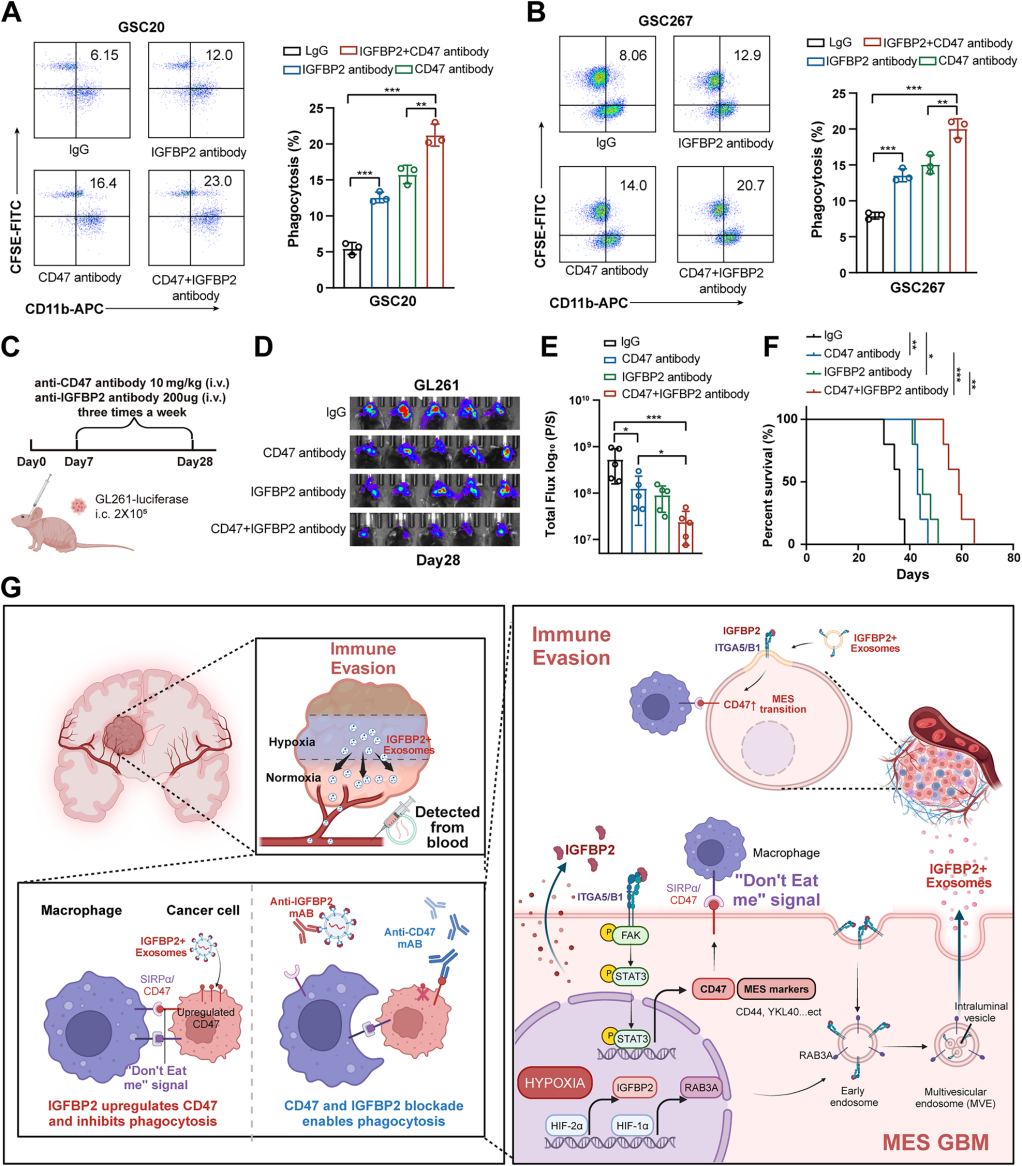
Combinatorial blockade of IGFBP2 and CD47 synergistically suppressed GBM immune evasion and malignant progression. **A**, **B** Flow cytometry analysis and quantification of macrophages phagocytosis in GSC20 and GSC267 cells treated with IgG or blocking antibody anti-CD47 and anti-IGFBP2. Right graph: Quantification of the percentage of macrophages phagocytosis. (*n* = 3) **C** Schematic representation of the homologous models established to evaluate the response to anti-CD47 and anti-IGFBP2 therapy. **D** Bioluminescence imaging of xenograft models established with GSC267 cells treated with anti-CD47 and anti-IGFBP2 antibody on the indicated days after surgery. **E** In vivo tumor activities were assessed by bioluminescent in vivo imaging system on the indicated days after surgery. (*n* = 5) **F** Kaplan-Meier survival curves for animals in different groups, *n* = 5 for each group. **G** A proposed model of hypoxia-exosomal IGFBP2-CD47 axis in the regulation of GBM immune evasion. Data are presented as the mean ± SD. Statistical significance was determined using one-way ANOVA and the log-rank test (*P < 0.05; **P < 0.01; ***P < 0.001).

Next, we further explored the role of targeting IGFBP2 in enhancing the therapeutic efficacy of anti-CD47 treatment. We treated the GSCs using anti-IGFBP2 and anti-CD47 antibodies and the results showed that combination of anti-IGFBP2 antibody and anti-CD47 antibody significantly promoted macrophages phagocytosis (Fig. 7A, B). Moreover, GL261 cells were implanted into mouse brains (4-week-old C57 mice) to generate orthotopic xenografts. And in vivo experiments revealed that combined anti-IGFBP2 and anti-CD47 treatment suppressed tumor growth and prolonged the survival time of tumor-bearing mice (Fig. 7C–F). Immunohistochemical analysis confirmed that the IGFBP2 antibody significantly reduced CD47 expression (Fig. S7A). Further experiments demonstrated that depletion of macrophages attenuated the therapeutic efficacy of combined anti-IGFBP2 and anti-CD47 antibody treatment, indicating that the therapeutic effect primarily depends on enhancing macrophage-mediated phagocytosis (Fig. S7B–D). Collectively, our data suggested that combinatorial blockade of IGFBP2 and CD47 synergistically suppressed GBM immune evasion and malignant progression, highlighting the therapeutic promise of this strategy in GBM.

## Discussion

CD47 is frequently overexpressed in GBM cells, playing a pivotal role in immune evasion by suppressing macrophage-mediated tumor cell clearance [41, 42]. The glioma microenvironment, characterized by its immunosuppressive nature, further amplifies CD47’s role in promoting tumor progression [42, 43]. Targeting CD47 has shown promise in preclinical studies, as it not only enhances the combination of anti-IGFBP2 antibody and anti-CD47 antibody significantly inhibited but also reprograms the tumor microenvironment to favor anti-tumor immunity. However, the heterogeneity of GBM and the potential for immune resistance highlight the need for combinatorial therapeutic approaches to fully exploit the potential of CD47 in GBM treatment. In this work, we analyzed scRNA-seq data and identified a close association between CD47 and the hypoxic microenvironment in GBM. It is reported that lactate producted by GBM cells and microglia/macrophages in hypoxic microenvironment induces tumor cell epigenetic reprogramming through histone lactylation that leads to suppression of phagocytosis via transcriptional upregulation of CD47 [9]. In our study, further experiments confirmed that hypoxia-induced IGFBP2 could bind to ITGA5/B1 and activate the FAK-STAT3 signaling pathway, which promotes the expression of CD47 and ultimately enhances immune escape in GBM. Moreover, The combination of anti-IGFBP2 and anti-CD47 antibodies could enhance the therapeutic efficacy of CD47-targeted treatment in glioma. Overall, this work identified IGFBP2 as a potential biomarker for predicting the efficacy of anti-CD47 therapy in GBM patients, providing a new strategy for combination therapy to reduce anti-CD47 resistance in GBM.

Recent studies have highlighted the significant role of exosomes in modulating the immune response, particularly in protein secretion [44]. These vesicles deliver secreted proteins to target cells in several mechanisms, thus influencing various physiological processes. First, exosomes directly package secreted proteins via endocytosis, incorporating them into their lumen [12]. This process ensures that the secreted proteins are protected from degradation in the extracellular environment. In addition to serving as cargo carriers, exosomes also mediate secreted proteins signaling through their surface receptors [24, 25, 45]. Exosome membranes display a range of receptors, such as integrins and tetraspanins, which can bind to secreted proteins and form stable complexes [24]. For example, cytokines such as CCL2 can interact with specific receptors to directly modulate the functional activity of target cells [25]. In our study, We observed that IGFBP2 is predominantly localized on the exosome surface through its interaction with ITGA5/B1. Upon internalization of the exosomes, the IGFBP2-ITGA5/B1 complex directly fuses with the target cell membrane, thereby triggering the activation of the downstream FAK/STAT3 signaling pathway.

In our study, we were excited to find that anti-IGFBP2 antibodies and anti-CD47 antibodies demonstrated combinatorial benefit in orthotopic glioma mouse models, but we recognize the limitations of this combination. Because targeting IGFBP2 downregulated the expression of CD47 on tumor cells, the CD47 antibody might lose enough targets, thereby resulting in a partial attenuation of the CD47 antibody’s therapeutic efficacy. Therefore, when combining CD47 and IGFBP2 antibodies, a carefully optimized regimen is necessary to ensure that their actions are complementary rather than mutually antagonistic. Moreover, CD47 antibody therapy can cause hematotoxicity, especially anemia, owing to the presence of CD47 on erythrocyte surfaces [46, 47]. The combination of CD47 antibody with IGFBP2 antibody enables a reduction in the dosage of CD47 antibody, thereby mitigating associated adverse effects.

Our findings highlight a potential role of hypoxia-induced IGFBP2 in regulating CD47 expression and macrophage phagocytosis. While CD47 has been identified as a direct HIF-1α target, suggesting multiple regulatory pathways under hypoxia, IGFBP2 likely serves as a complementary mechanism that reinforces CD47 induction and immune evasion. The impact of IGFBP2-containing exosomes on CD47 expression and macrophage activity in non-hypoxic tumor regions remains an exciting area for future in vivo investigation. Approaches such as spatial transcriptomics or in situ hypoxia labeling may further clarify the coordinated roles of IGFBP2 and HIF-1α in shaping an immunosuppressive microenvironment.

## Supplementary information

Supplementary figures

Supplementary methods.

Table S1. The list of primer sequences

Table S2. Sequences for siRNAs X shRNAs.

Table S3. The detailed antibody information.

Table S4. Mass spectrometry results of the potential proteins that bind to IGFBP2.

uncropped western blots

## Data Availability

All data are included within the main text and supplementary files. Protein spectrum results are available in Supplementary Files. Further inquiries can be directed to contact the corresponding authors.
