# Supplementary material for: Targeting hypoxic exosomal IGFBP2 overcomes CD47-mediated immune evasion in glioblastoma
Source: Cell Death Dis. 2026 Jan 31;17(1):192. doi: 10.1038/s41419-026-08430-9 (PMC12876975; doi:10.1038/s41419-026-08430-9)
Supplement: Supplementary file 1 — Supplementary figures [file 41419_2026_8430_MOESM1_ESM.docx]

**Supplementary Figures and Figure Legends for**

**Targeting hypoxic exosomal IGFBP2 overcomes CD47-mediated**

**immune evasion in glioblastoma**

Yanhua Qi^1,2#^, Rongrong Zhao^1,2#^, Xinglong Zhang^1,2^, Huize Xia^1,2^, Ping Zhang^1,2^, Qingtong Wang^1,2^, Shulin Zhao^1,2^, Shaobo Wang^1,2^, Xiaofan Guo^1,2,4^, Wei Qiu^1,2^, Boyan Li^1,2^, Ziwen Pan^1,2^, Jiawei Qiu^1,2^, Zijie Gao^1,2^, Haiquan Lu^3*^, Gang Li^1,2*^, Hao Xue^1,2*^

**Affiliations:**

1. Department of Neurosurgery, Qilu Hospital, Cheeloo College of Medicine and Institute of Brain and Brain-Inspired Science, Shandong University, Jinan 250012, Shandong, P. R. China.

2. Shandong Key Laboratory of Brain Health and Function Remodeling, Jinan 250012, P. R. China.

3. Advanced Medical Research Institute and Key Laboratory for Experimental Teratology of the Ministry of Education, Cheeloo College of Medicine, Shandong University, Jinan, Shandong 250012, China.

4. Department of Neurology, UPMC Stroke Institute, University of Pittsburgh School of Medicine, Pittsburgh, Pennsylvania, USA

# These authors contributed equally to this work.

**Correspondence:**

1. Hao Xue, Department of Neurosurgery, Qilu Hospital, Cheeloo College of Medicine and Institute of Brain and Brain-Inspired Science, Shandong University, Jinan 250012, Shandong, P. R. China. Tel/Fax: +86-18560089163; E-mail: xuehao@sdu.edu.cn;

2. Gang Li, Department of Neurosurgery, Qilu Hospital, Cheeloo College of Medicine and Institute of Brain and Brain-Inspired Science, Shandong University, Jinan 250012, Shandong, P. R. China. Tel/Fax: +86-18560085699; E-mail: dr.ligang@sdu.edu.cn

3. Haiquan Lu, Advanced Medical Research Institute and Key Laboratory for Experimental Teratology of the Ministry of Education, Cheeloo College of Medicine, Shandong University, Jinan, Shandong 250012, China. E-mail: [lvhaiquan@sdu.edu.cn](mailto:lvhaiquan@sdu.edu.cn)


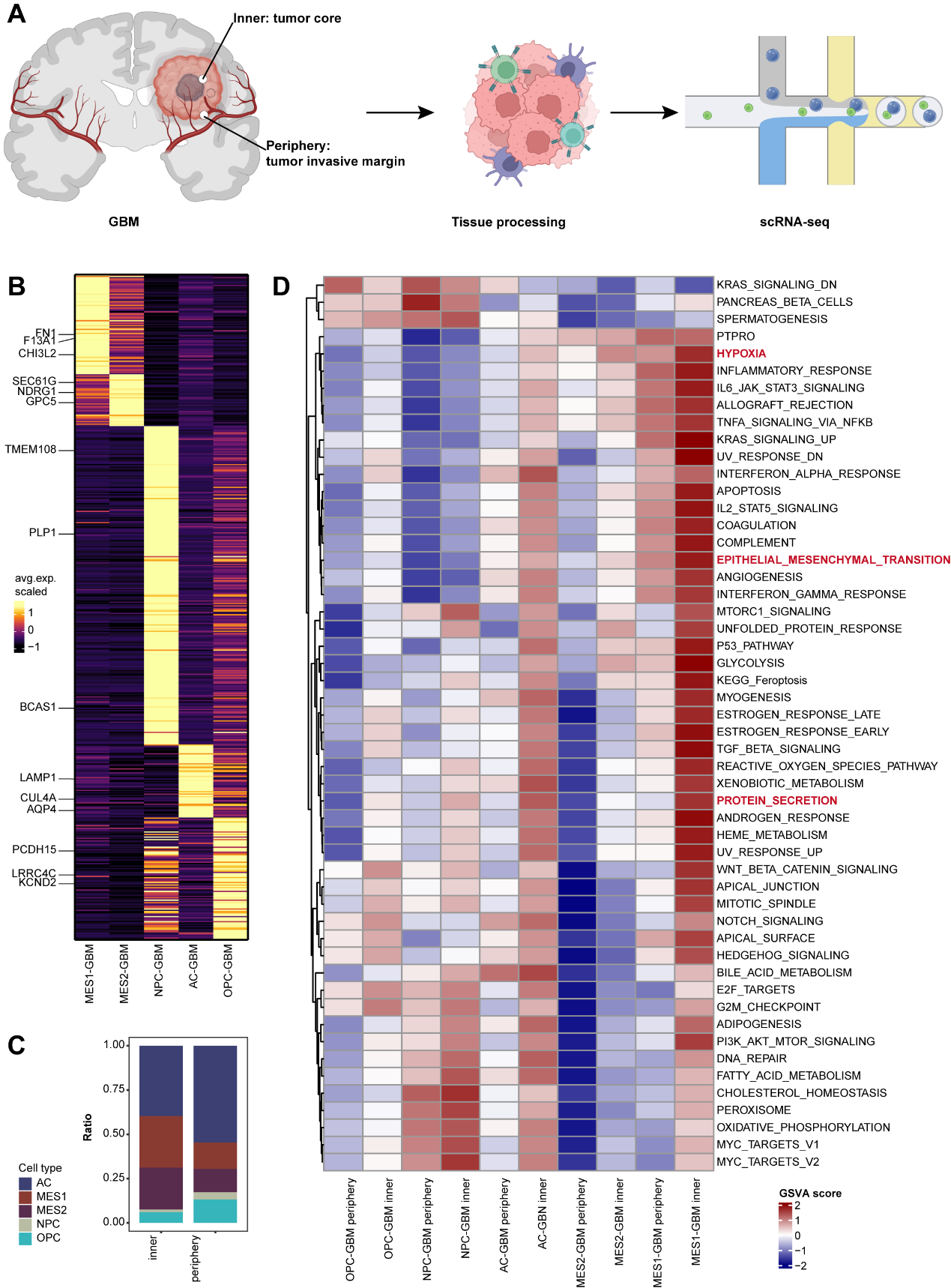


**Figure S1 Single-cell sequencing data divides tumor cells into five major subtypes.**

**A.** Schematic overview of single-cell RNA-seq (scRNA) with the 10×Genomics Chromium System on GBM tissue isolated from GBM patients. Tissues at distinct locations (Inner: tumor core; Periphery: tumor invasive margin) were collected.

**B.** Heatmap showing four major malignant subtypes, including neural progenitor-like GBM cells (NPC-GBM), oligodendrocyte progenitor-like GBM cells (OPC-GBM), astrocytic-like GBM cells (AC-GBM), as well as mesenchymal-like GBM cells (MES-GBM), which was further divide into MES1-GBM and MES2-GBM subpopulations. **C.** Bar plots showing the percentage of each celltype in scRNA-seq. **D.** Heatmap showing pathway activity differences scored per cell by GSVA across malignant subpopulations.


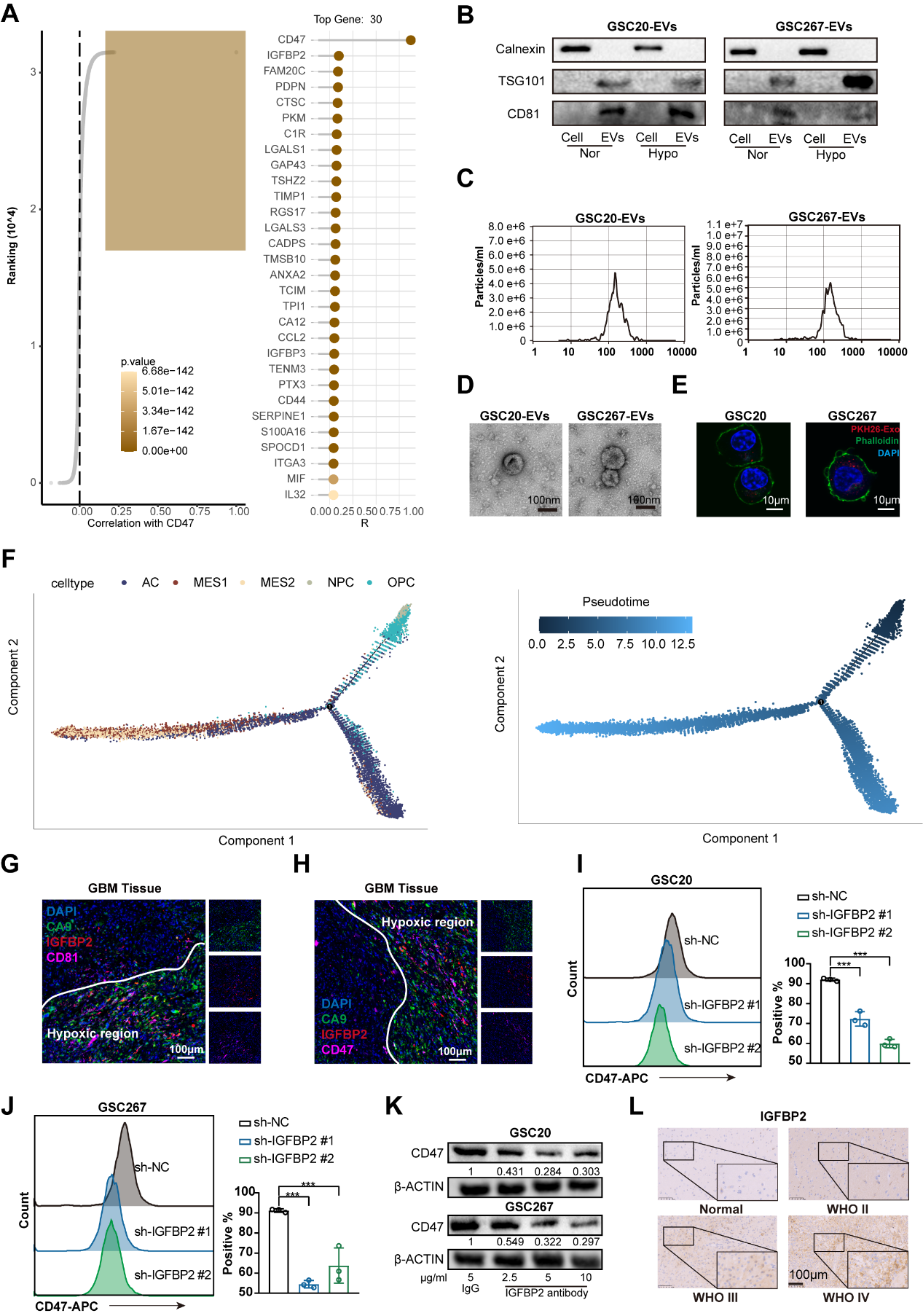


**Figure S2 IGFBP2 is upregulated in GBM patients and indicates a poor prognosis of GBM.**

**A.** Genes were positively associated with CD47. **B.** Western blot analysis of the presence of TSG101 and CD81 and the absence of calnexin in N-GDEs and H-GDEs. **C.** NanoSight particle tracking analysis of the size distributions and number of exosomes. **D.** Representative transmission electron micrograph of exosomes isolated from culture supernatants of the glioma cell lines GSC20 and GSC267 (Scale bar, 100 nm). **E.** GSC cells were incubated with PKH26-labeled GSC-derived exosomes for 24 h. Scale bar, 10 nm **F.** Monocle2 analysis revealing that MES state was gradually evolved with tumor progression. **G.** Immunofluorescence staining shows that IGFBP2-positive exosomes are highly enriched in the hypoxic regions of GBM patient tissues, with CA9 indicating hypoxia and CD81 serving as an exosomal marker. Scale bar, 100μm. **H.** Immunofluorescence staining shows that IGFBP2 and CD47 are co-localized in the hypoxic regions of GBM patient tissues, as indicated by CA9. Scale bar, 100μm. **I-J.** Flow cytometry analysis and quantification of CD47 expression on GSC20 and GSC267 cells transfected with sh-NC or sh-IGFBP2. Right graph: Quantiﬁcation of the percentage of CD47^+^ cells. (n =3). **K.** Western blot assay showing the expression of CD47 in GSC cells treated with IgG or IGFBP2 antibody. **L.** Representative IHC-staining images showing a significant increase in IGFBP2 expression in tumor tissues of high-grade glioma patients. Scale bar, 100μm.Data are presented as the mean ± SD. Statistical significance was determined using one-way ANOVA (*P < .05; **P < .01; ***P < .001).


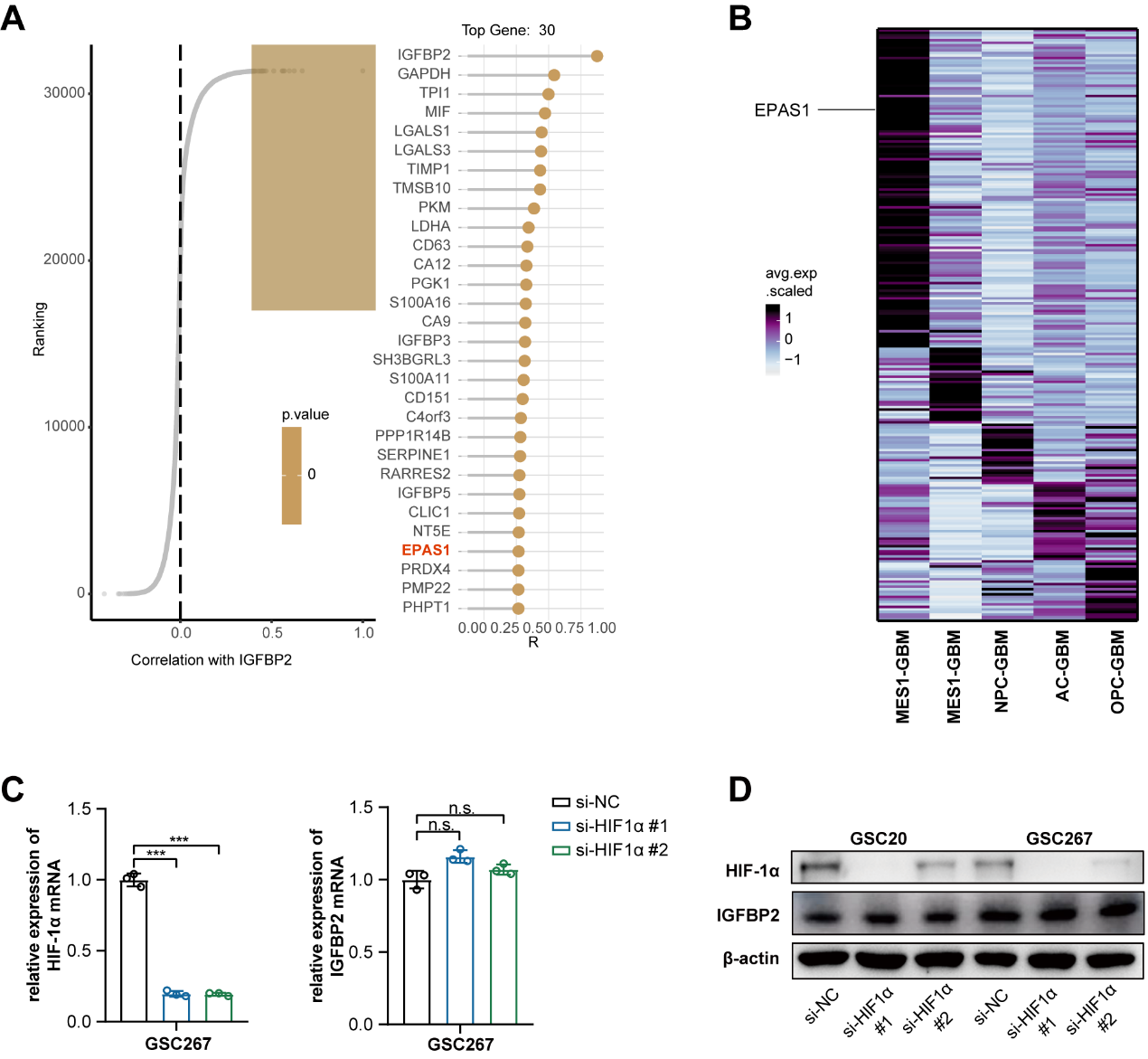
**Figure S3 HIF-2α upregulates IGFBP2 under hypoxia. A.** Intersection analysis of the top 30 genes positively correlated with IGFBP2. **B.** Heatmap of enriched TFs in NPC-like, OPC-like, AC-like, MES1-like and MES2-like GBM cells subtypes. **C-D.** qPCR assay and western blot assay showing the expression of HIF-1α and IGFBP2 treated with si-NC or si-HIF-1α. Data are presented as the mean ± SD. Statistical significance was determined using one-way ANOVA (*P < .05; **P < .01; ***P < .001).

**
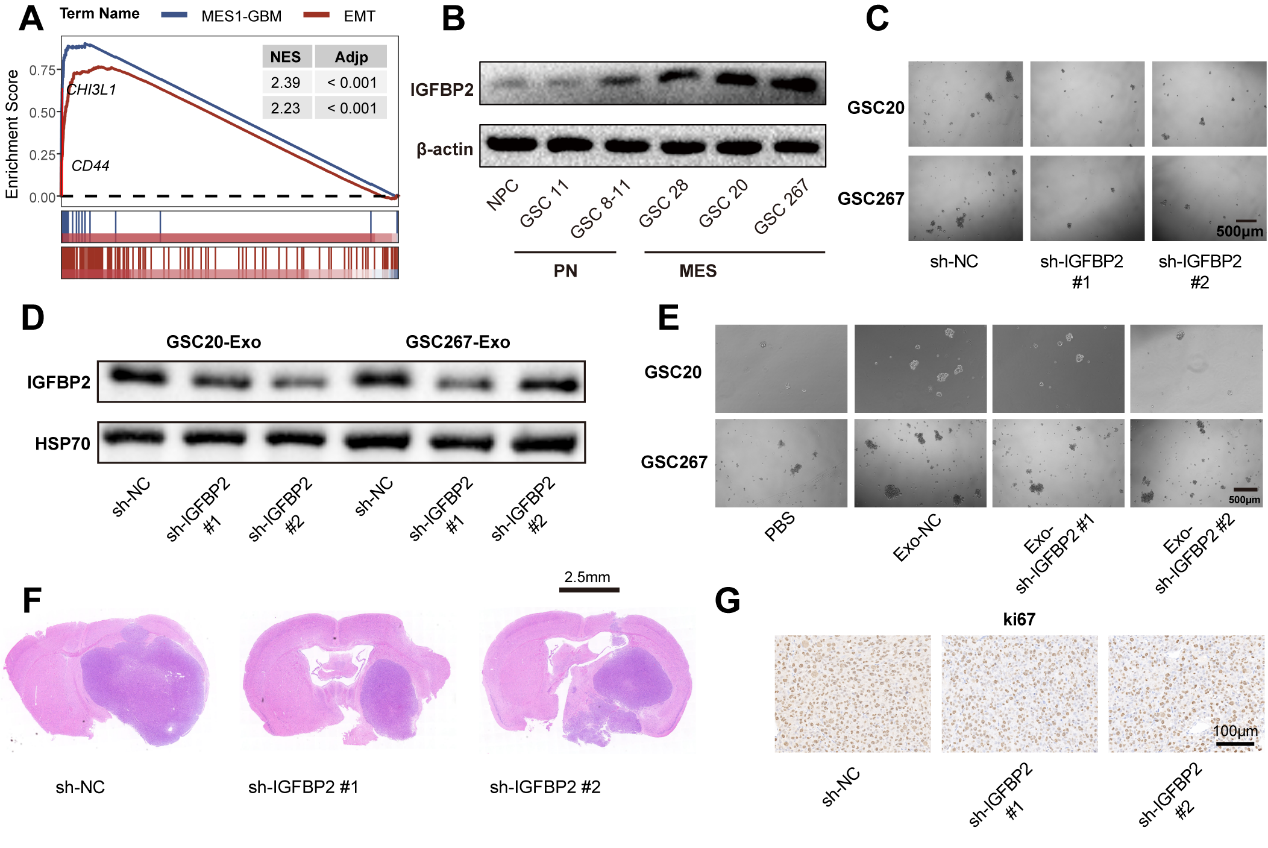
**

**Figure S4 Exosomal IGFBP2 promotes MES transformation and tumorigenesis in GBM.**

**A.** ScRNA GSEA analysis showed that genes positively associated with IGFBP2 were significantly enriched in MES1 and EMT enrichment. **B.** Western blot assay showing the expression of IGFBP2 in PN-subtype and MES-subtype GSCs. **C.** Quantified analysis of spheres diameter of tumor spheres formation after GSC20 and GSC267 cells treated with exosome derived from the cell supernatant. **D.** Western blot assay showing the expression of IGFBP2 in exosomes of GSC20 and GSC267 transfected with sh-NC or sh-IGFBP2. **E.** Quantified analysis of spheres diameter of tumor spheres formation after GSC20 and GSC267 cells treated with exosome derived from the cell supernatant. **F.** Representative image of H&E staining in different group. **G.** Representative IHC-staining images showing the relative expression of ki67 in IGFBP2 knockdown orthotopic xenograft models. Scale bar, 100μm. Data are presented as the mean ± SD. Statistical significance was determined using one-way ANOVA (*P < .05; **P < .01; ***P < .001).


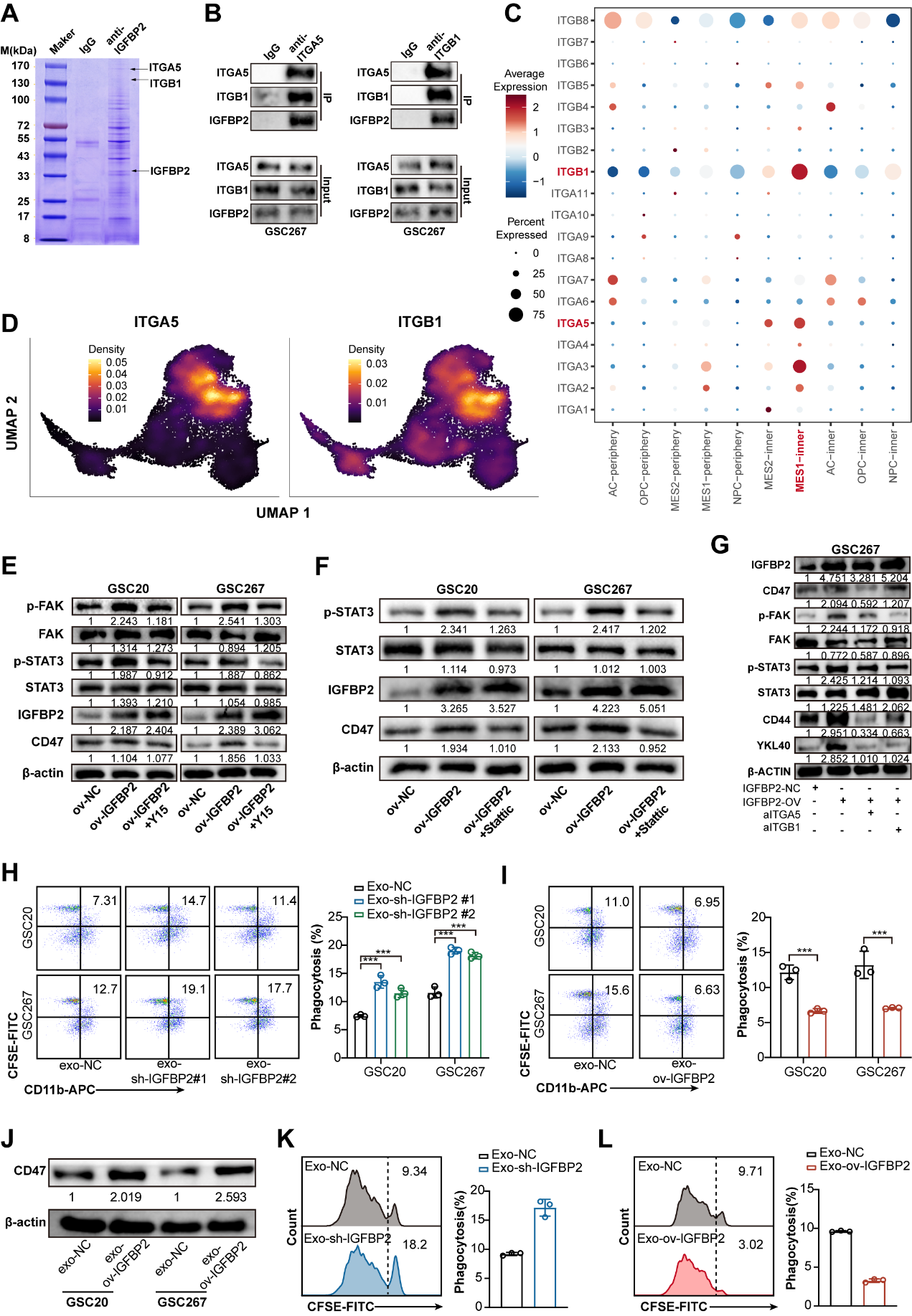


**Figure S5 Exosomal IGFBP2 interacts with integrin α5β1 to upregulates CD47 expression via FAK-STAT3 signaling. A.** Coomassie blue staining assays showing the proteins that interacted with IGFBP2, which were identified by co-IP/mass spectrometry; arrows indicate IGFBP2 and integrin α5β1 protein bands. **B.** Co-IP and western blot assays showing the interaction of IGFBP2, ITGA5 and ITGB1 using anti-ITGA5 and anti-ITGB1 antibody. **C.** Bubble plot indicating that ITGA5 and ITGB1 were highly expressed in the MES1-inner subpopulation. **D.** Umap and density plots showing that ITGA5 and ITGB1 were expressed in the MES1-like GBM subpopulation. **E, F.** Western blot assays showing the protein expression of FAK-STAT3 signaling pathway and CD47 in GSC20 and GSC267 cells transfected with ov-NC or ov-IGFBP2 and treated with specific STAT3 inhibitors (Stattic, 5 μM) and FAK inhibitors (Y15, 10μM). **G.** Western blot assays showing the protein expression of FAK-STAT3 signaling pathway, MES phenotype markers (CD44 and YKL40) and CD47 in GSC20 cells with transfected with ov-NC or ov-IGFBP2 and treated with IgG or blocking antibody anti-integrin α5β1. **H-I.** Flow cytometry analysis and quantification of macrophages phagocytosis in GSC20 and GSC267 cells treated with exosome derived from the cell supernatant. Right graph: Quantiﬁcation of the percentage of macrophages phagocytosis. (n =3) **J.** Western blot assays showing the protein expression of CD47 in GSC20 and GSC267 cells treated with exosome derived from the cell supernatant. **K-L.** Flow cytometry analysis and quantification of macrophages phagocytosis *in vivo* treated with exosome derived from the cell supernatant. Right graph: Quantiﬁcation of the percentage of macrophages phagocytosis. (n =3) Data are presented as the mean ± SD. Statistical significance was determined using one-way ANOVA (*P < .05; **P < .01; ***P < .001).


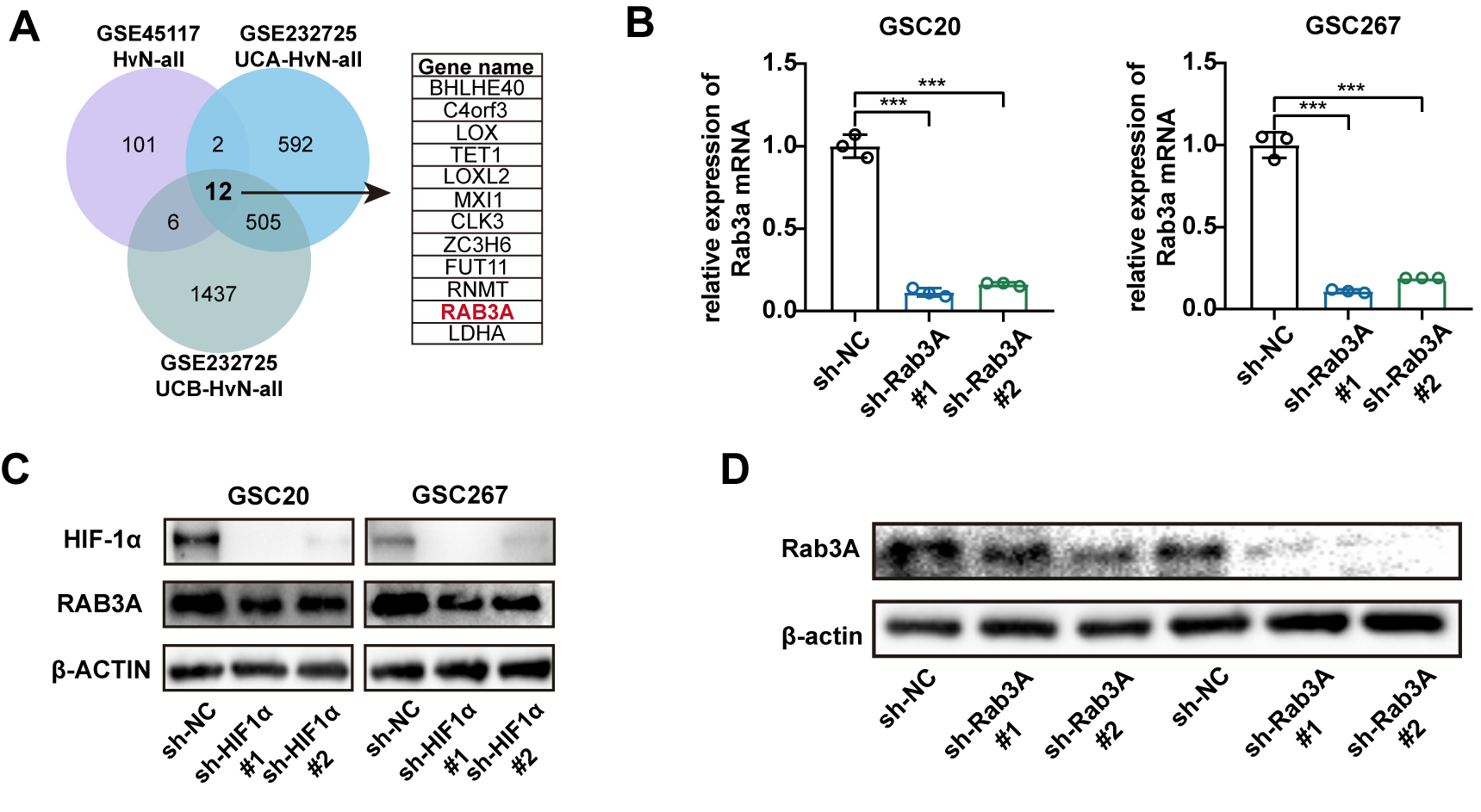


**Figure S6 RAB3A is upregulated to increase the secretion of exosomes under hypoxia. A.** RNA sequencing of GSE45117 and GSE232725 showing the upregulation of RAB3A under hypoxia. **B, C.** qPCR assay and western blot assay showing the expression of RAB3A in GSC20 and GSC267 cells transfected with sh-NC or sh-RAB3A. **D.** Western blot analysis of exosomes puriﬁed from equal numbers of GSC20 and GSC267 cells transfected with sh-NC or sh-RAB3A. Exosomes were collected from 20 × 10^6^ cells for each group. Data are presented as the mean ± SD. Statistical significance was determined using one-way ANOVA (*P < .05; **P < .01; ***P < .001).


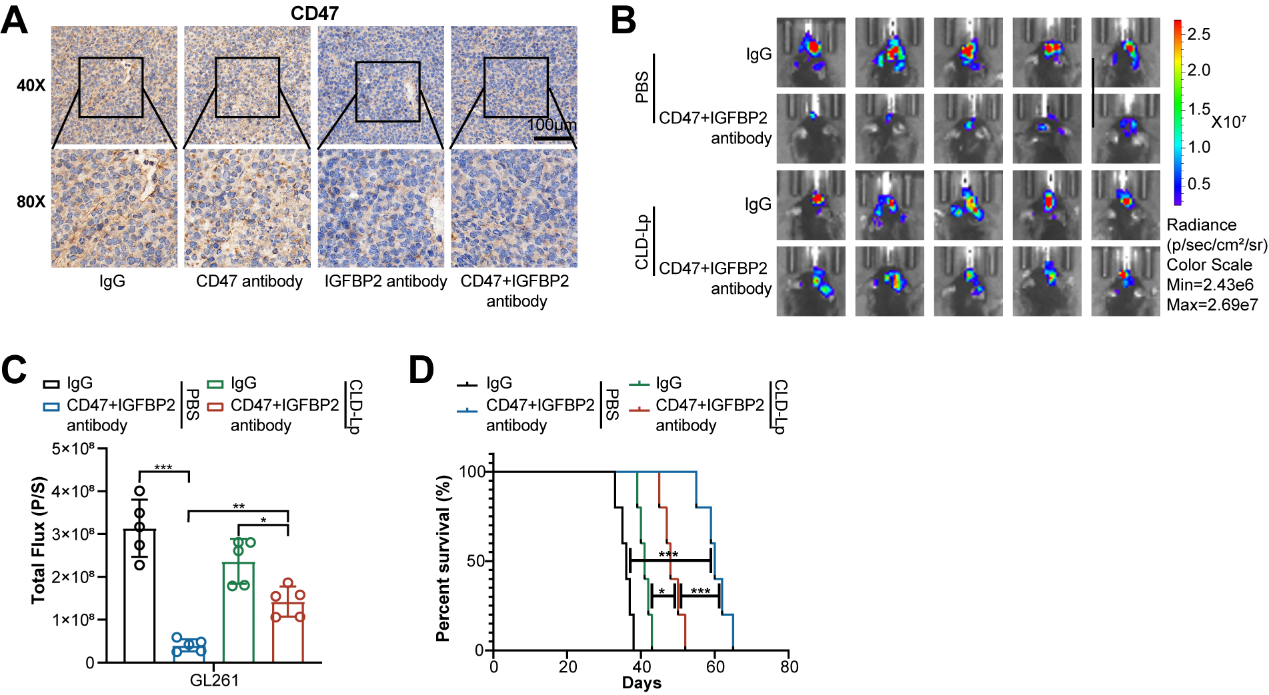


**Figure S7 Combinatorial blockade of IGFBP2 and CD47 synergistically suppressed GBM immune evasion and malignant progression. A.** Representative IHC-staining images showing CD47 expression of tumor tissues in different groups. Scale bar, 100μm. **B, C.** Bioluminescence imaging of xenograft models established with GL261 cells in different groups. In vivo tumor activities were assessed by bioluminescent in vivo imaging system. n = 5. **D.** Kaplan-Meier survival curves for animals in different groups, n = 5 for each group. Data are presented as the mean ± SD. Statistical significance was determined using one-way ANOVA (*P < .05; **P < .01; ***P < .001).
