# Supplementary material for: Targeting hypoxic exosomal IGFBP2 overcomes CD47-mediated immune evasion in glioblastoma
Source: Cell Death Dis. 2026 Jan 31;17(1):192. doi: 10.1038/s41419-026-08430-9 (PMC12876975; doi:10.1038/s41419-026-08430-9)
Supplement: Supplementary file 2 — Supplementary methods. [file 41419_2026_8430_MOESM2_ESM.docx]

**Supplementary Methods**

**Exosome isolation and identification**

The culture medium from glioma cells and human plasma (3ml) was collected and subjected to a series of centrifugation steps: 300g for 10 minutes, 2000g for 30 minutes, and 12,000g for 45 minutes. The resulting supernatant was filtered through a 0.22 μm membrane and ultracentrifuged at 110,000g for 70 minutes. The pellet was washed with PBS and centrifuged again at 110,000g for 70 minutes. The purified exosomes were then resuspended in PBS and stored at -80°C. Transmission electron microscopy (HT-7700, Hitachi) was employed to observe the morphology and size of the exosomes, while their size and concentration were measured using the ZetaView system (Particle Metrix, Germany).

**In vitro and in vivo experiments with exosome treatment**

For in vitro experiments, recipient cells were treated with exosomes at a final concentration of 1 μg/mL (quantified based on total exosomal protein) for 48 h. Experimental mice were intravenously injected with exosomes (100 μg/mouse) isolated from the 48h culture supernatants. Mice received three injections of the exosomes per week for 3 weeks.

**Western blotting**

Cellular and exosomal proteins were extracted using RIPA buffer supplemented with a protease inhibitor cocktail (Sigma-Aldrich). Protein concentrations were determined using a BCA Protein Assay Kit (Beyotime, China). The proteins were resolved by SDS-PAGE and transferred onto polyvinylidene difluoride (PVDF) membranes. Details of the primary antibodies used are provided in Table S3.

**Transfection of small interfering RNA (siRNA) and plasmid**

siRNA, plasmid, and corresponding negative control (NC) were obtained from Boshang Biotechnology Co., Ltd. (Shanghai, China). siRNA and plasmid transfections were performed using the Lipofectamine 3000 kit (#L3000015, Invitrogen, Carlsbad, CA, USA). Cells transfected with sh-IGFBP2 and sh-RAB3A were subjected to puromycin selection to establish stable cell lines. Sequences are provided in Supplementary Table S2.

**RNA extraction and real-time PCR analysis**

RNA was extracted from cells using the RNA-Quick Purification Kit (RN001, ES Science) following the manufacturer’s instructions. Reverse transcription was performed using the ReverTra Ace qPCR RT Kit (FSQ-101, TOYOBO). Quantitative PCR was carried out using TB Green™ Premix Ex Taq™ (Takara).β-actin served as internal controls for mRNA. The primer sequences are provided in Table S1.

**Luciferase reporter assay**

After seeded onto 24-well plates for 24 h, the 293T cells were transfected with 0.75 μg pGL3-basic-IGFBP2 promoter-luciferase reporter and 0.25 μg HIF-2α expression plasmid or empty vector along with 0.1 μg pRL-TK for normalization. Then the luciferase activity was measured using the Dual Luciferase Reporter Assay System (Beyotime, China) according to the manufacturer’s instructions.

**Immunofluorescence**

For cell immunofluorescence assays, cells were cultured in confocal dishes with indicated treatment, and then fixed with 4% paraformaldehyde. After washed with PBS and penetrated with 0.5% Triton X-100, samples were blocked with 5% BSA and incubated with indicated primary antibodies overnight at 4◦C. Then samples were stained with Alexa Fluor 594-conjugated secondary antibodies (Yeasen, China, 1:200) for 1 h at RT. Cytoskeleton was visualized by FITC-phalloidin (Yeasen, China, 1:200) staining and nuclei was visualized by 4′,6-diamidino-2-phenylindole (DAPI; Yeasen, China, 1:5000) staining. Images were obtained with Leica SP8 confocal microscope.

**Flow cytometry analysis**

In vitro phagocytosis assays were performed using human monocyte-derived THP1 cells differentiated into macrophages by incubation with 100 nM Phorbol 12-myristate 13-acetate (PMA) for 48 hours. Human GBM cell lines were labeled with carboxyfluorescein diacetate succinimidyl ester (CFSE, Cat# V12883, Invitrogen, USA). Labeled GBM cells (1 × 10⁶) were cocultured with macrophages (1 × 10⁶) in six-well plates for 24 hours at 37 °C. Following coculture, cells were collected, washed with 0.5% BSA-PBS, and incubated with fluorescent APC-CD11b primary antibody (Catalog# 101225, BioLegend) in the dark for 30 minutes. After three additional washes with 0.5% BSA-PBS, the phagocytic activity was analyzed by flow cytometry.

For the evaluation of CD47, GSC cells were incubated with anti-CD47 eFluor 710 (46-5983-42, eBioscience) at RT for 30min. Flow cytometry was performed on BD Accuri C6 flow cytometer platform. Data were analyzed with FlowJo V10 software.

**Tumor sphere formation assay**

Glioma stem cells (GSCs) were seeded into 6-well plates at a density of 1,000 cells per well and cultured for 1 to 2 weeks in 1.5 mL of culture medium. The relative diameters of the tumor spheres were measured and analyzed using a Leica DMi8 microscope for quantification.

**Extreme limiting dilution assay (ELDA)**

GSCs were seeded into ultralow-attachment 96-well plates at varying cell densities: 1, 2, 4, 8, 16, 32, 64, and 128 cells per well, with 10 replicates for each density. After seven days of incubation, the number of wells exhibiting tumor sphere formation was recorded. The data were analyzed using ELDA software (<http://bioinf.wehi.edu.au/software/elda/>).

**Co-immunoprecipitation assay**

The Pierce Direct Magnetic IP/Co-immunoprecipitation (Co)-IP Kit (88828, Thermo Fisher Scientific) was used following the manufacturer’s instructions. In brief, cells were collected and treated with IP lysis buffer mixed with protease inhibitor cocktails. Anti-IGFBP2 (ab188200, Abcam) and isotype IgG (2729, Cell Signaling Technology) were added to the cell lysates and rotated at 4°C overnight. Next, magnetic beads were added to each sample and then incubated at 37°C for 2 h. The proteins were isolated, boiled with SDS loading buffer, and analyzed through mass spectrometry and western blotting.

**GBM-brain organoid co-culture invasion**

The GBM-brain organoid co-culture invasion ex vivo system, such as the culture of 18-day rat fetal brain organoids, was performed as previously described (Bjerkvig R, Laerum O, Mella O. Glioma cell interactions with fetal rat brain aggregates in vitro and with brain tissue in vivo. Cancer Res. 1986;46:4071–9). GFP-transfected GBM cells were cultured to generate glioma spheroids and then co-cultured with mature brain organoids for 72 h. GBM cell invasion images were captured under confocal microscopy (Leica TCS SP8; Wetzlar, Germany).

**Single cell data preprocess**

Raw FASTQ files were mapped to the Reference genome (GRCh38/hg38) using Cell Ranger 3.0 (10x Genomics). To create Cell Ranger-compatible reference genomes, the references were rebuilt according to instructions from 10x Genomics, which were performed alignment, filtering, barcode counting and UMI counting. Following alignment, digital gene expression (DGE) matrices were generated for each sample and for all samples. Merged 10x Genomics DGE files were generated using the aggregation function of the Cell Ranger pipeline. All the cells in different batches were merged together normalized by equalizing the read depth among libraries. The final result was the matrix of all cells and their global gene expressions.

**UMAP visualization and determination of the major cell types**

Gene expression analysis and cell type identification was analyzed using Seurat V2.0 pipeline (http://satijalab.org/seurat/) after filtering and normalization, another R toolkit for single-cell transcriptomics (Butler et al., 2018). As the data were already normalized, they were loaded into Seurat without normalization, scaling or centring. Along with the expression data, metadata for each cell was collected, including information location. Next, highly variable genes were identified and used as input for dimensionality reduction via principal component analysis (PCA). The resulting PCs and the correlated genes were examined to determine the number of components to include in downstream analysis. UMAP was then performed on the first 20 principal components to visualize cells in a two-dimensional space. To identify differentially expressed genes in each cluster, the Seurat function FindAllMarkers was used. For a gene to be differentially expressed in a cluster it must be expressed by at least 10% of cells, have a log-fold change greater than 0.25, and reach statistical significance of an adjusted p < 0.05 as determined by the Wilcox test. Finally, cell clusters were annotated to known biological cell types using canonical marker.

**Pseudotime Analysis**

Single cell trajectory was analyzed using matrix of cells and gene expressions by Monocle 2(1). Differentially expressed genes or significantly variable genes among cells were identified and used for dynamic trajectory analysis which ordered cells in pseudotime. First, the expression of transcripts of each gene was determined. Genes were then ranked using the coefficient of variation versus mean metric, selecting the top 20 genes for each celltype as features. The resulting velocity estimates were projected onto the UMAP embedding obtained in Seurat.

**Functional analysis of differentially expressed genes (DEGs)**

DEG analysis across all cell types was carried out using the Wilcoxon test integrated into the Seurat package. To estimate pathway activity at the single-cell level, the Gene set variation analysis (GSVA) package (version 1.22.4) was employed with default settings(2). Gene Set Enrichment Analysis (GSEA) was conducted using version 4.0.3 of the GSEA software, which incorporates predefined gene sets from the Molecular Signatures Database (MSigDB v7.1, https://www.gsea-msigdb.org/gsea/msigdb).

**Reference**

1. Qiu X, Mao Q, Tang Y, Wang L, Chawla R, Pliner HA*, et al.* Reversed graph embedding resolves complex single-cell trajectories. Nat Methods **2017**;14:979-82

2. Hanzelmann S, Castelo R, Guinney J. GSVA: gene set variation analysis for microarray and RNA-seq data. BMC Bioinformatics **2013**;14:7
