# Supplementary material for: Targeting hypoxic exosomal IGFBP2 overcomes CD47-mediated immune evasion in glioblastoma
Source: Cell Death Dis. 2026 Jan 31;17(1):192. doi: 10.1038/s41419-026-08430-9 (PMC12876975; doi:10.1038/s41419-026-08430-9)
Supplement: Supplementary file 3 — Table S1. The list of primer sequences [file 41419_2026_8430_MOESM3_ESM.docx]

**Table S1 The list of primer sequences**

| Name | Sequence (5’-3’) |
| --- | --- |
| Homo-IGFBP2-123F | ACAATGGCGATGACCACTCA |
| Homo-IGFBP2-123R | CCAGCTCCTTCATACCCGAC |
| Homo-RAB3A-93F | CGAGTTCTTTGAGGCAAGCG |
| Homo-RAB3A-93R | GACTCGGACATCTTCTCGCA |
| Homo-HIF1A-86F | GGCAGCAACGACACAGAAAC |
| Homo-HIF1A-86R | TTTTCGTTGGGTGAGGGGAG |
| Homo-HIF2A (EPAS1)-79F | TCATGGGACTTACACAGGTGG |
| Homo-HIF2A (EPAS1)-79R | CGAATCTCCTCATGGTCGCA |
| P1-141F(CHIP) | GGATTATTTTAGCGGGCGGAG |
| P1-141R(CHIP) | GCCCCGAATGGTCTTAGTGAC |
| P2-91F(CHIP) | CTCGCGAACTGAACTGAGAGC |
| P2-91R(CHIP) | GAGAATACGGATAACTCGCGG |
