# Supplementary material for: Targeting hypoxic exosomal IGFBP2 overcomes CD47-mediated immune evasion in glioblastoma
Source: Cell Death Dis. 2026 Jan 31;17(1):192. doi: 10.1038/s41419-026-08430-9 (PMC12876975; doi:10.1038/s41419-026-08430-9)
Supplement: Supplementary file 4 — Table S2. Sequences for siRNAs X shRNAs. [file 41419_2026_8430_MOESM4_ESM.docx]

**Table S2 Sequences for siRNAs & shRNAs**

| Name | Sequence (5’-3’) |
| --- | --- |
| sh-CTRL | 5'-UUCUCCGAACGUGUCACGUTT-3' |
| sh-IGFBP2#1 | 5'-CCAGTTCTGACACACGTATTT-3' |
| sh-IGFBP2#2 | 5'-ACAGTGCAAGATGTCTCTGAA-3' |
| si-HIF1α#1 | 5'-CAAUCAAGAAGUUGCAUUATT-3' |
| si-HIF1α#2 | 5'-UCGACUAUCUGCUCCAAGUUCTT-3' |
| si-HIF2α#1 | 5'-CUCCUCAGUUUGCUCUGAATT-3' |
| si-HIF2α#2 | 5'-CAGAACUGAUUGGUUACCATT-3' |
| sh-Rab3A#1 | 5'- GACCATCTATCGCAACGACAA-3' |
| sh-Rab3A#2 | 5'- CTACATGTTCAAGATTCTCAT-3' |
