# Supplementary material for: Targeting hypoxic exosomal IGFBP2 overcomes CD47-mediated immune evasion in glioblastoma
Source: Cell Death Dis. 2026 Jan 31;17(1):192. doi: 10.1038/s41419-026-08430-9 (PMC12876975; doi:10.1038/s41419-026-08430-9)
Supplement: Supplementary file 5 — Table S3. The detailed antibody information. [file 41419_2026_8430_MOESM5_ESM.docx]

**Supplementary Table 3 The detailed antibodies information**

| Antibodies | Brand | Catalogue |
| --- | --- | --- |
| CD47 | Cell Signaling Technology | 63000 |
| β-actin | Proteintech | 20536-1-AP |
| IGFBP2 | Abcam | ab188200 |
| HSP70 | Proteintech | 10995-1-AP |
| CD44 | Cell Signaling Technology | 37259 |
| YKL40 | Cell Signaling Technology | 47066 |
| ITGA5 | Cell Signaling Technology | 98204 |
| ITGB1 | Cell Signaling Technology | 34971 |
| FAK | Cell Signaling Technology | 3285 |
| p-FAK | Cell Signaling Technology | 3283 |
| STAT3 | Cell Signaling Technology | 9139 |
| p-STAT3 | Cell Signaling Technology | 9145 |
| HIF-1α | Proteintech | 20960-1-AP |
| HIF-2α | Proteintech | 26422-1-AP |
| CD81 | Proteintech | 66866-1-Ig |
| TSG101 | Proteintech | 28283-1-AP |
| Calnexin | Proteintech | 10427-2-AP |
| RAB3A | Proteintech | 15029-1-AP |
