# Supplementary material for: Targeting hypoxic exosomal IGFBP2 overcomes CD47-mediated immune evasion in glioblastoma
Source: Cell Death Dis. 2026 Jan 31;17(1):192. doi: 10.1038/s41419-026-08430-9 (PMC12876975; doi:10.1038/s41419-026-08430-9)

Figure 1 H

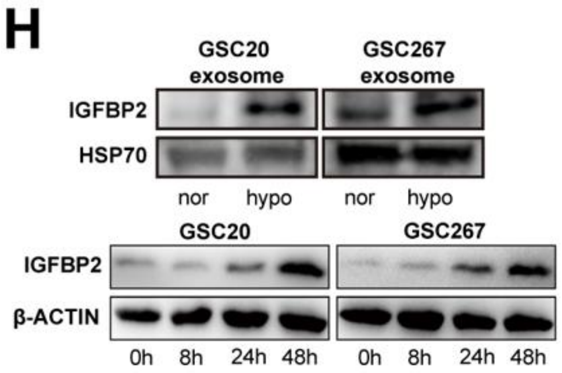

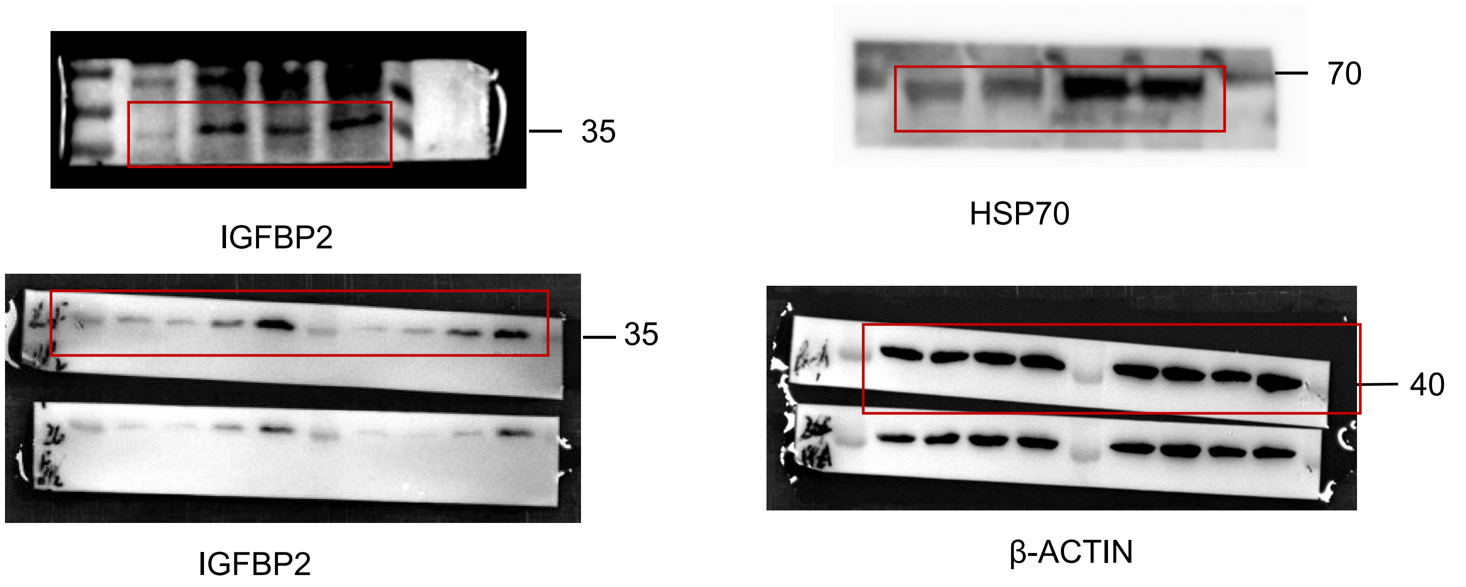

Figure 1I

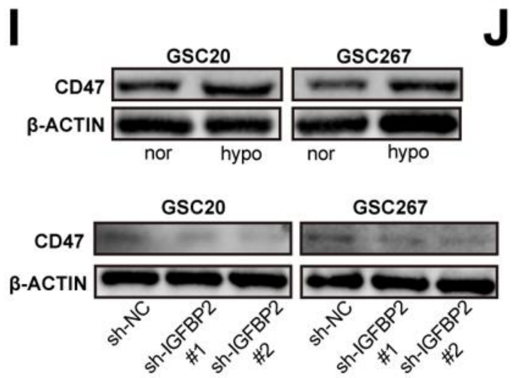

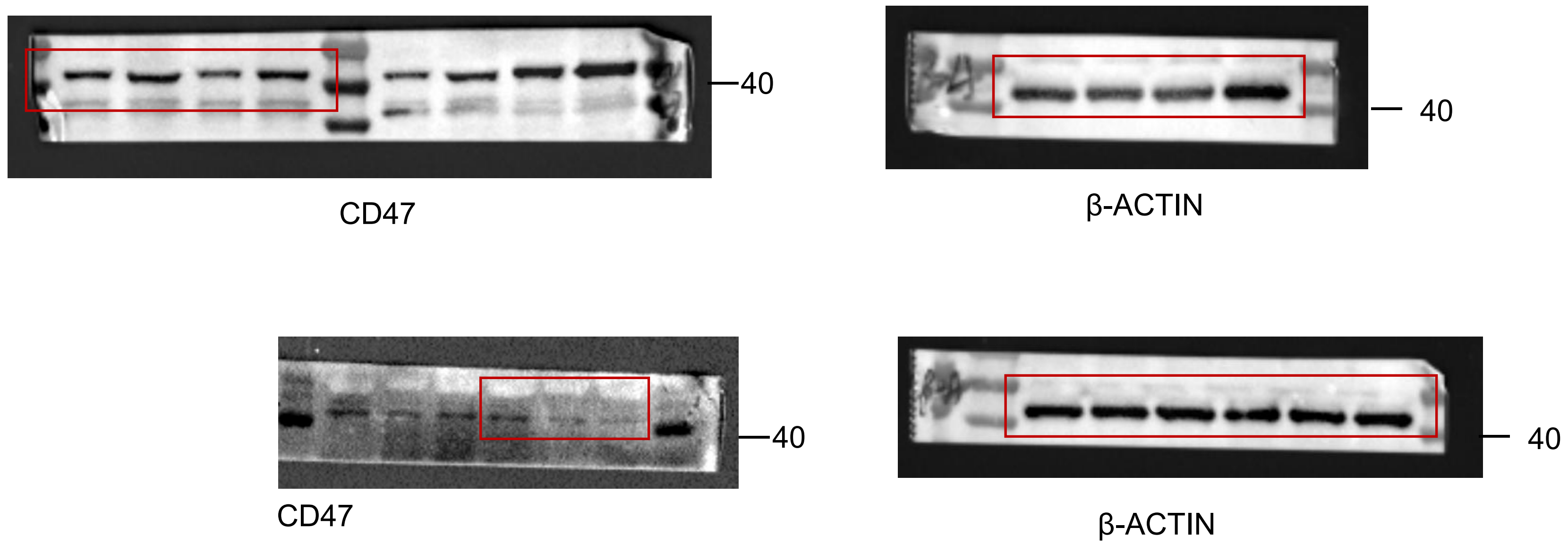

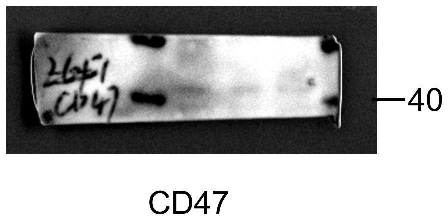

Figure 1J

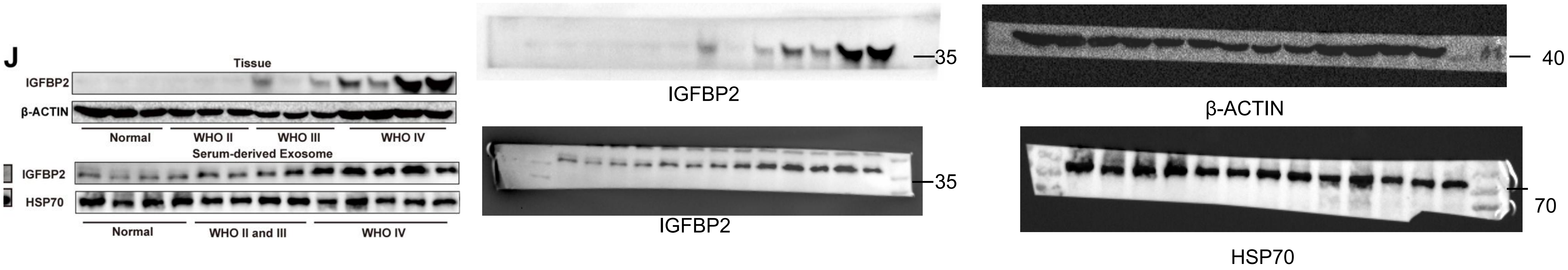

Figure 2E

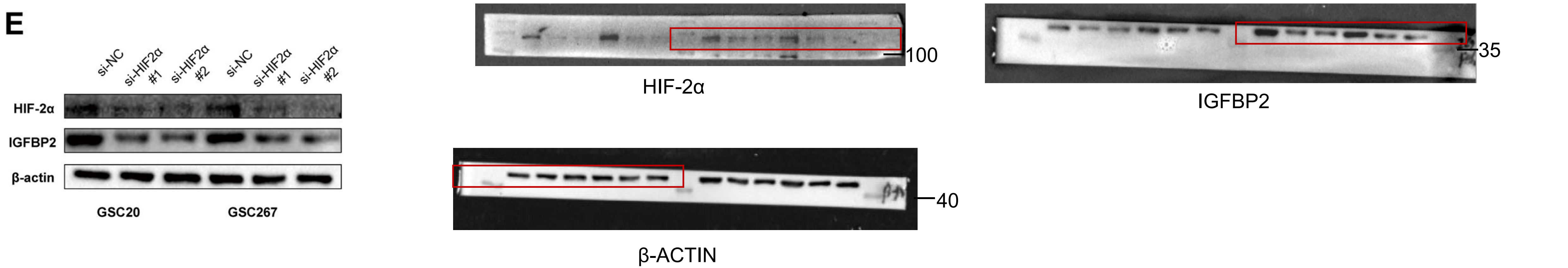

Figure 3A

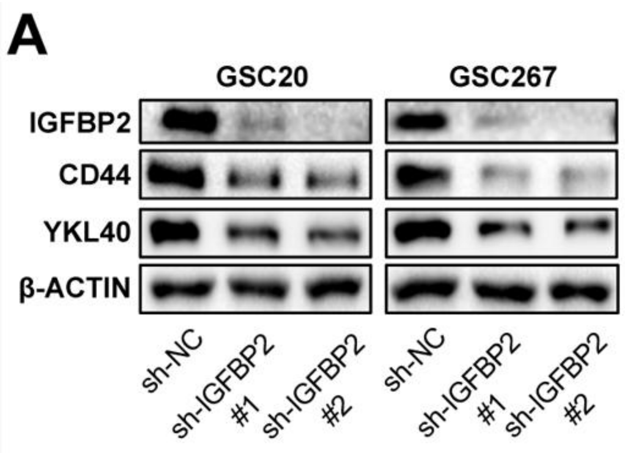

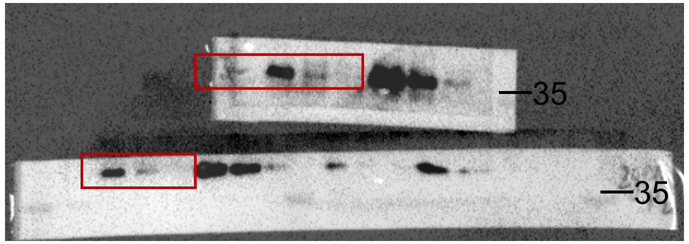

IGFBP2

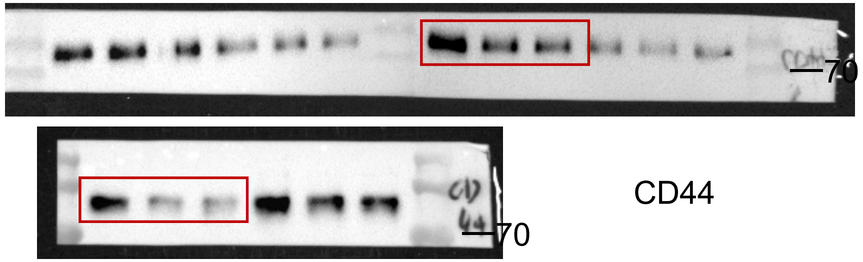

CD44

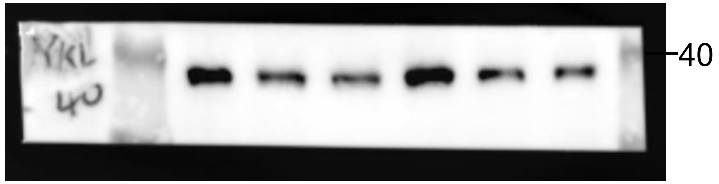

YKL40

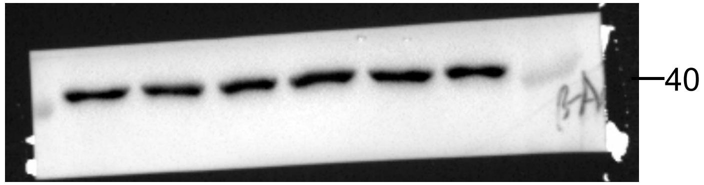

β-ACTIN

Figure 3F

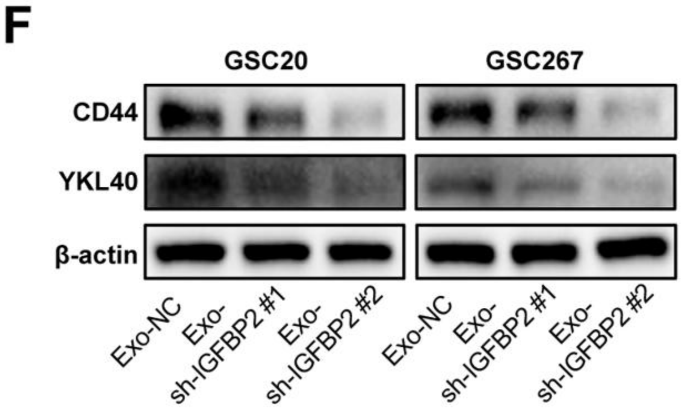

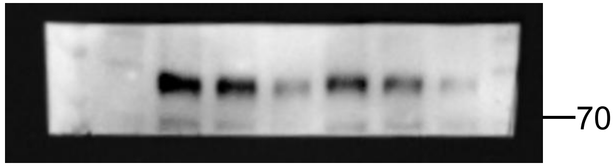

CD44

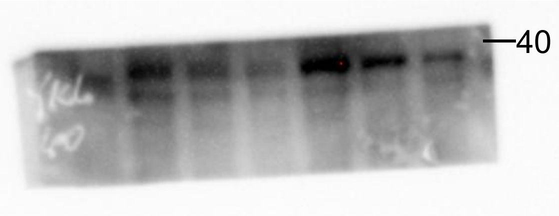

YKL40

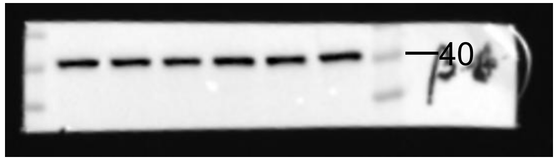

β-ACTIN

Figure 4B

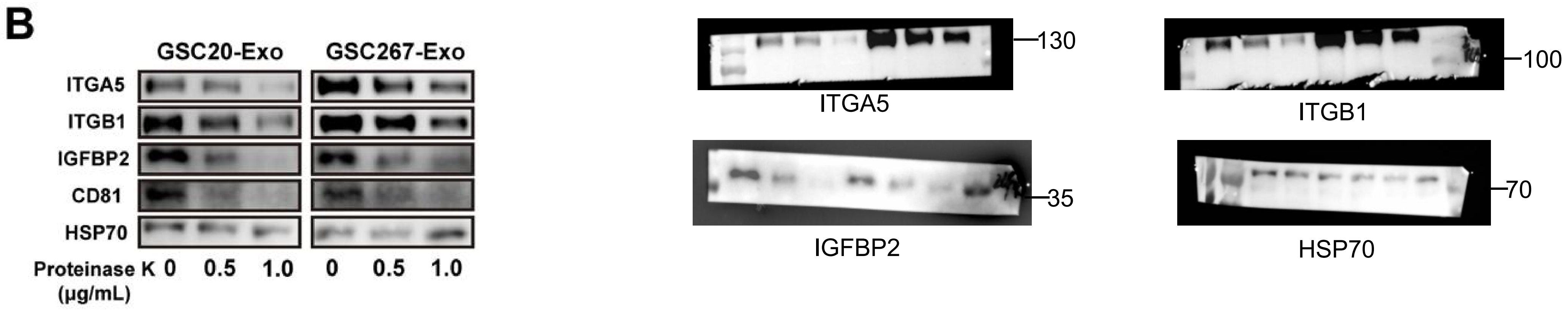

Figure 4C

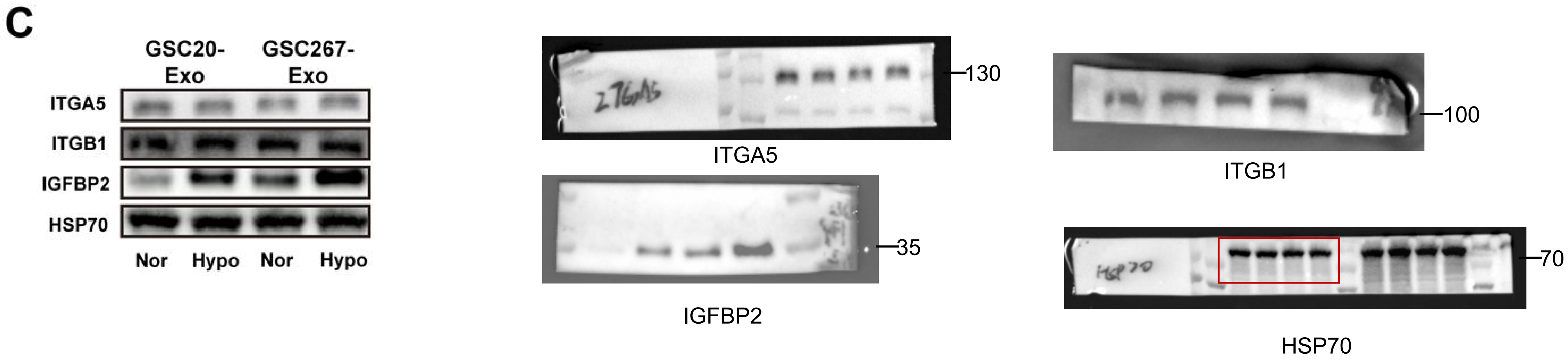

Figure 4F

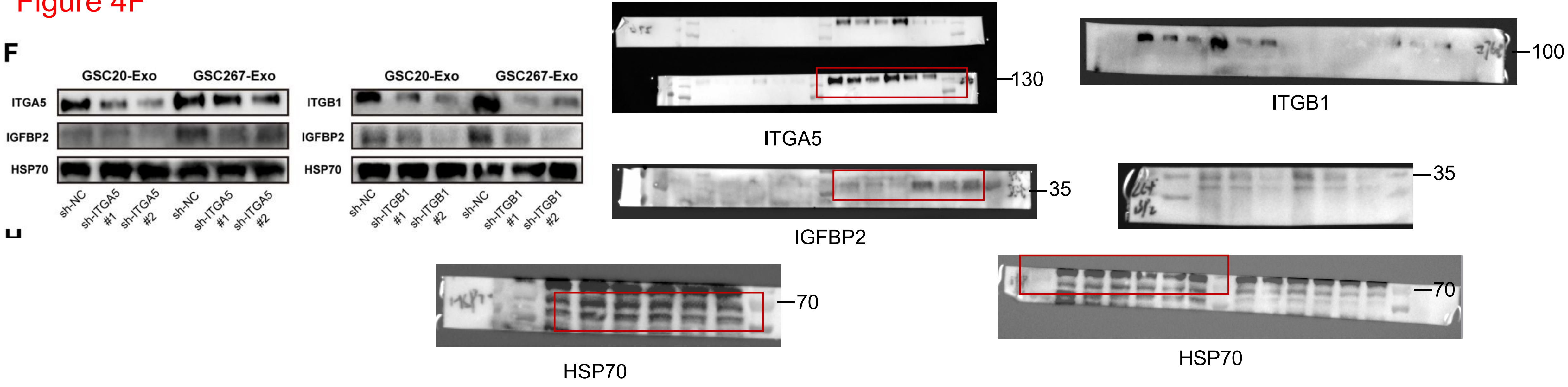

Figure 5B

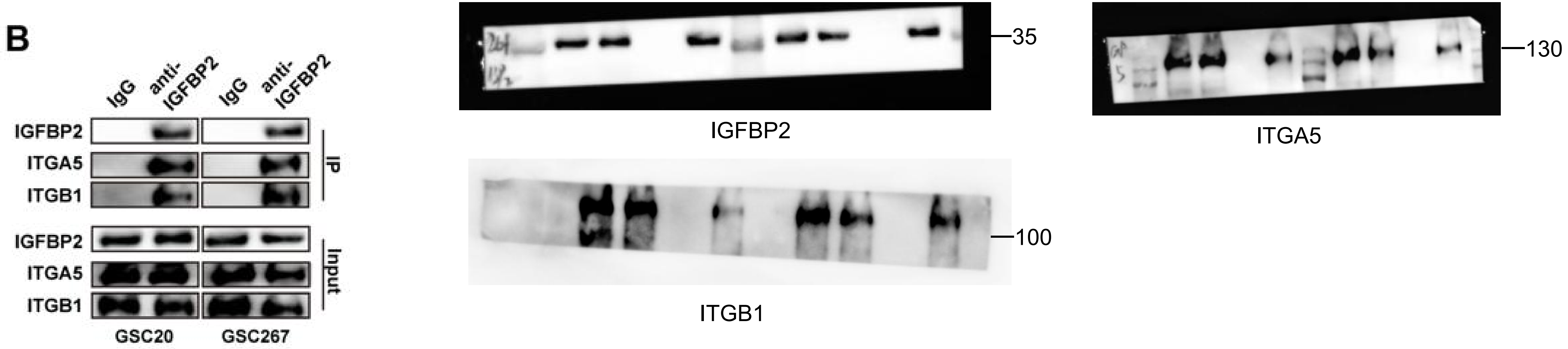

Figure 5D

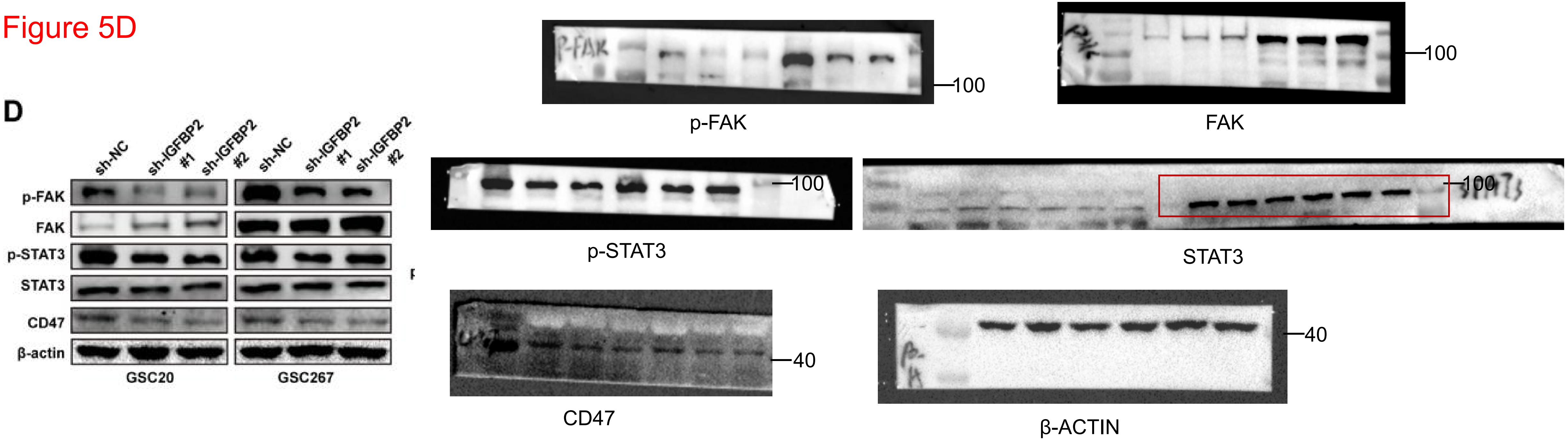

Figure 5E

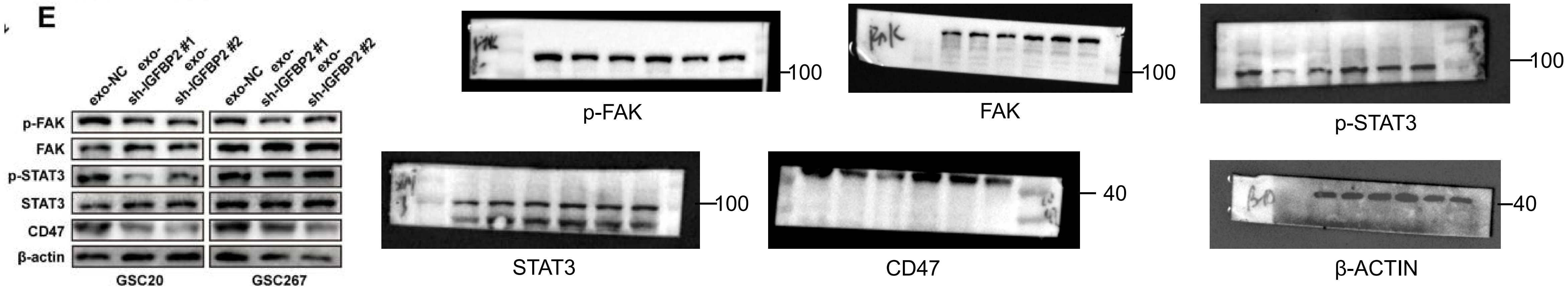

Figure 5F S5G

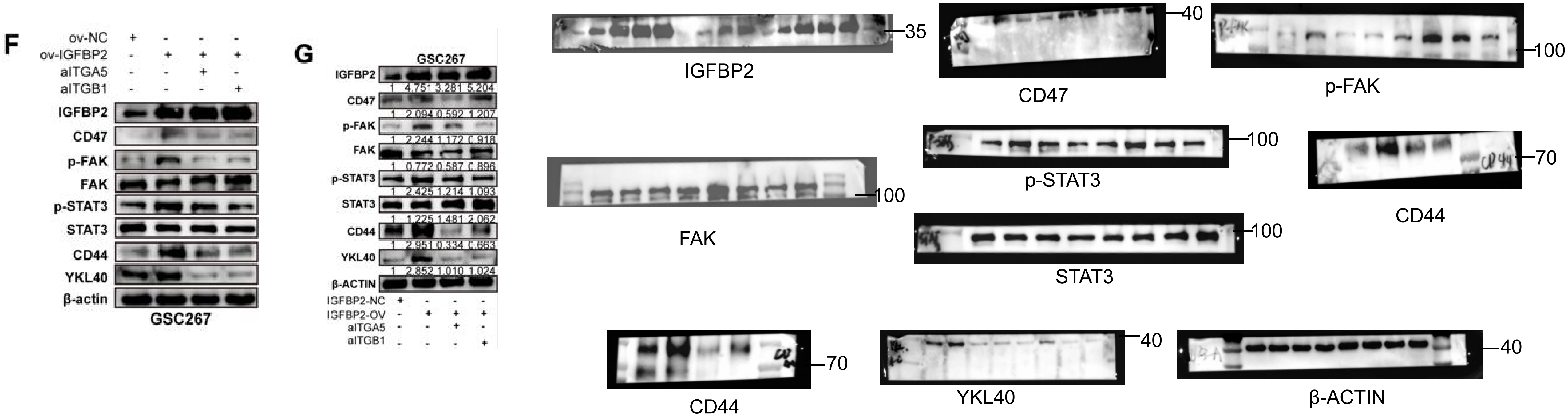

Figure 6B C

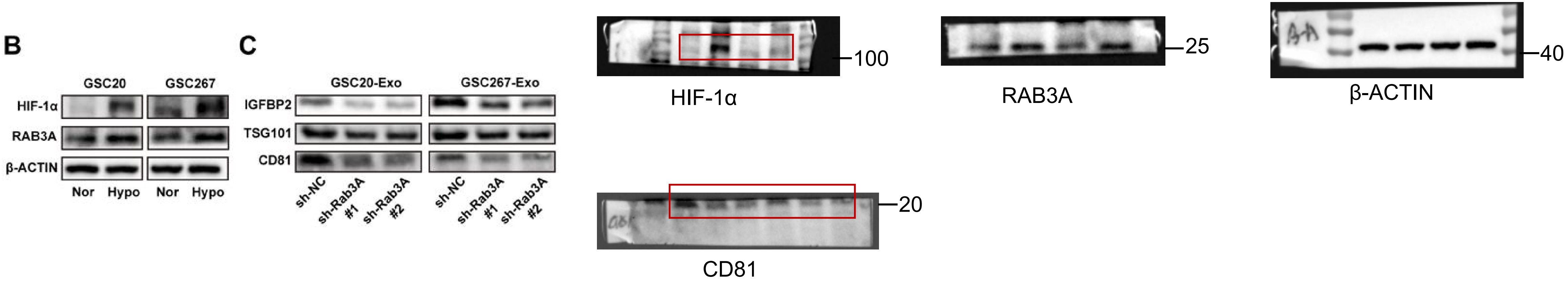

Figure S2B

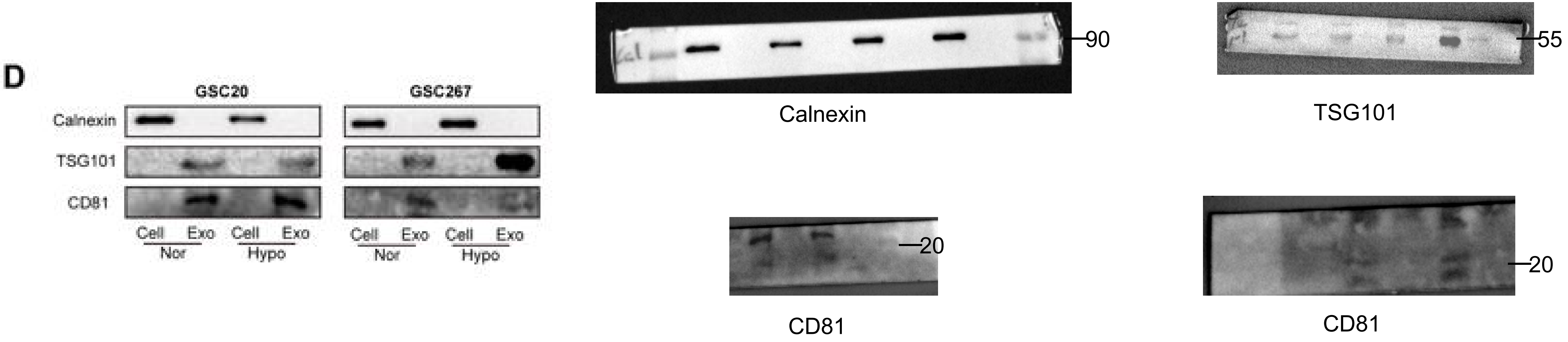

Figure S2K

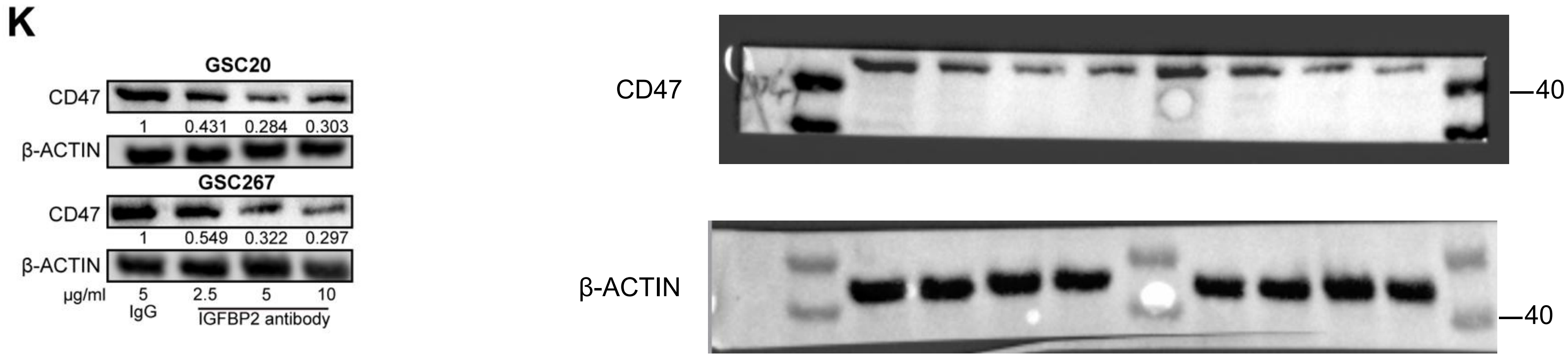

Figure S3D

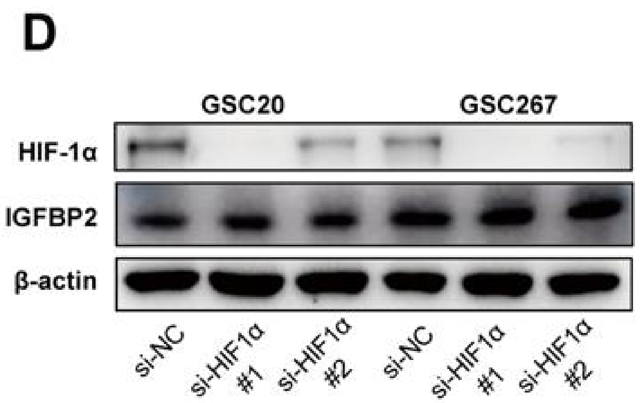

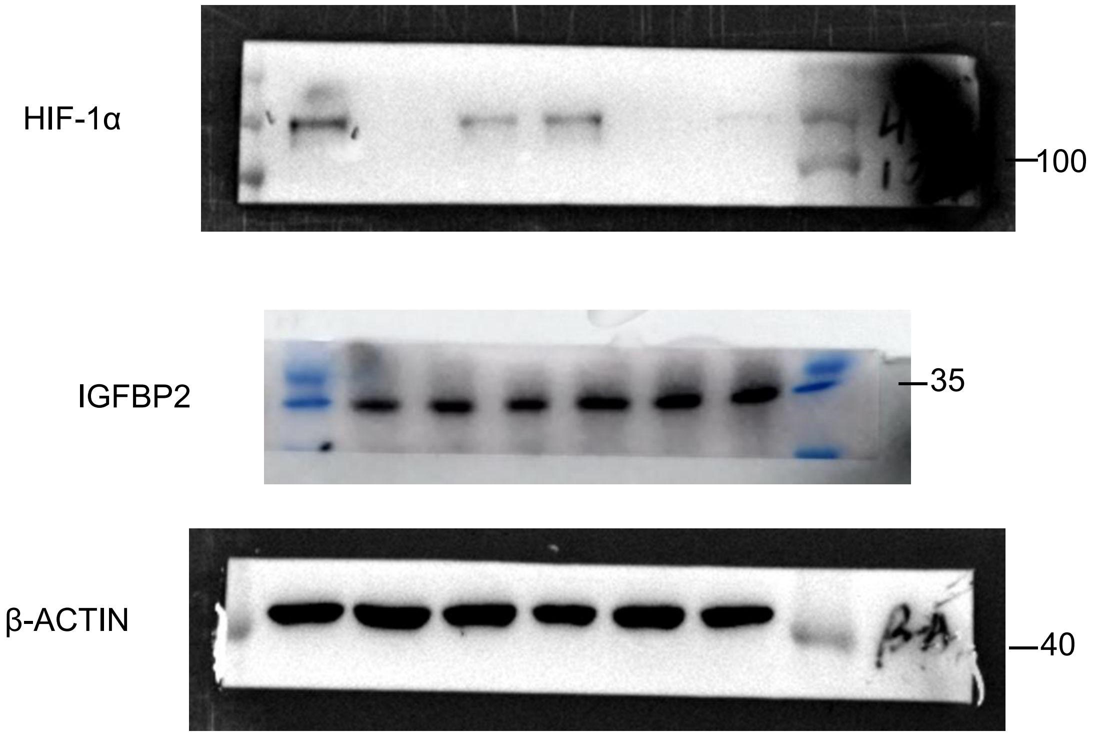

Figure S4B D

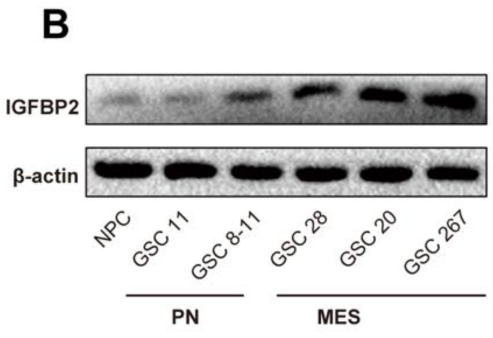

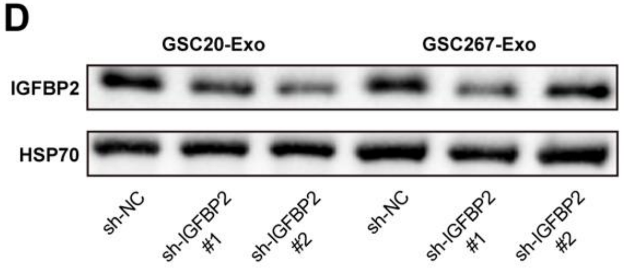

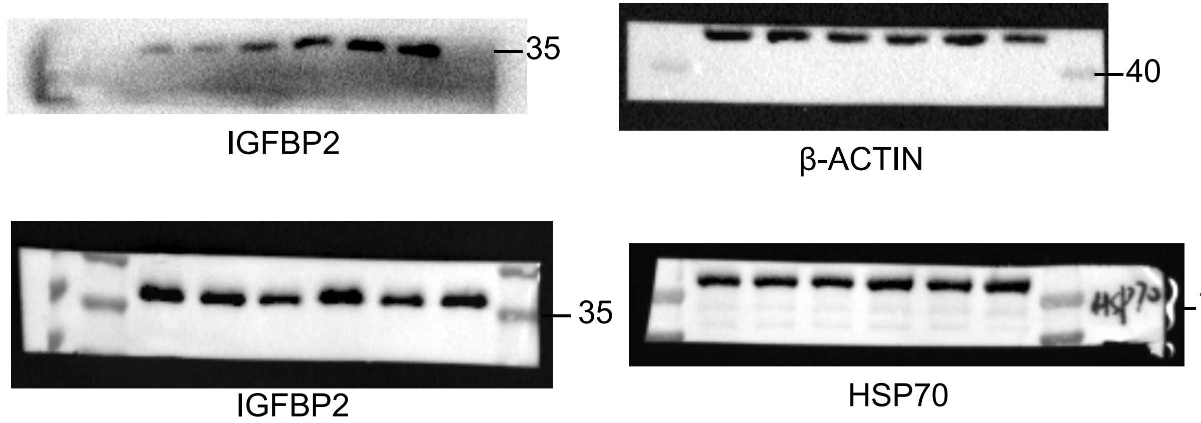

Figure S5B

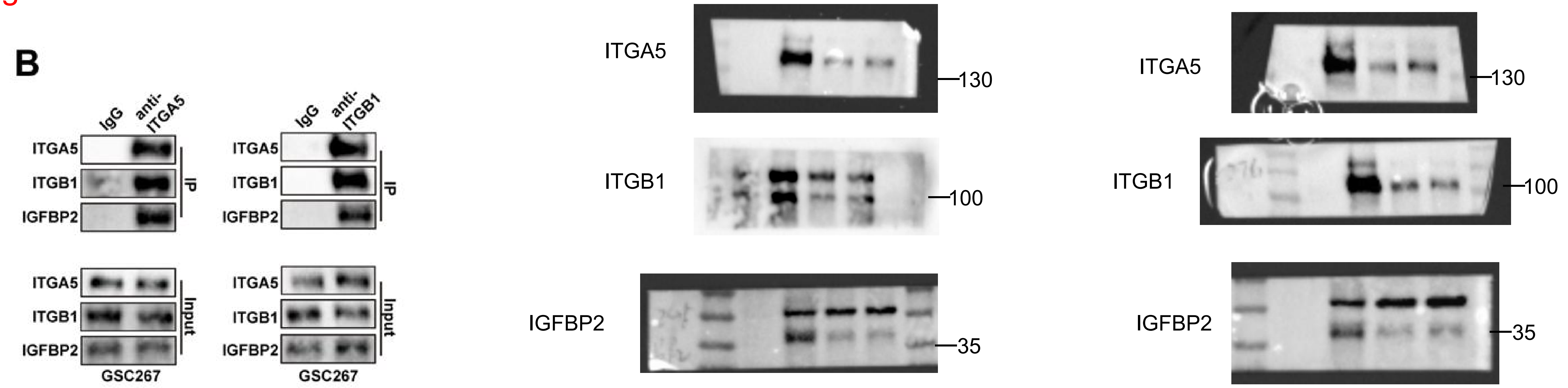

Figure S5E

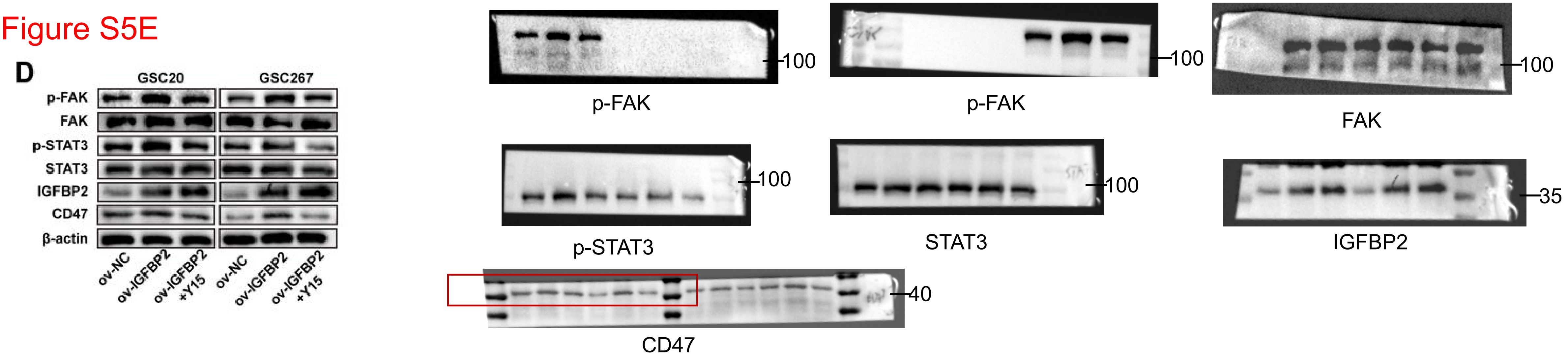

Figure S5F

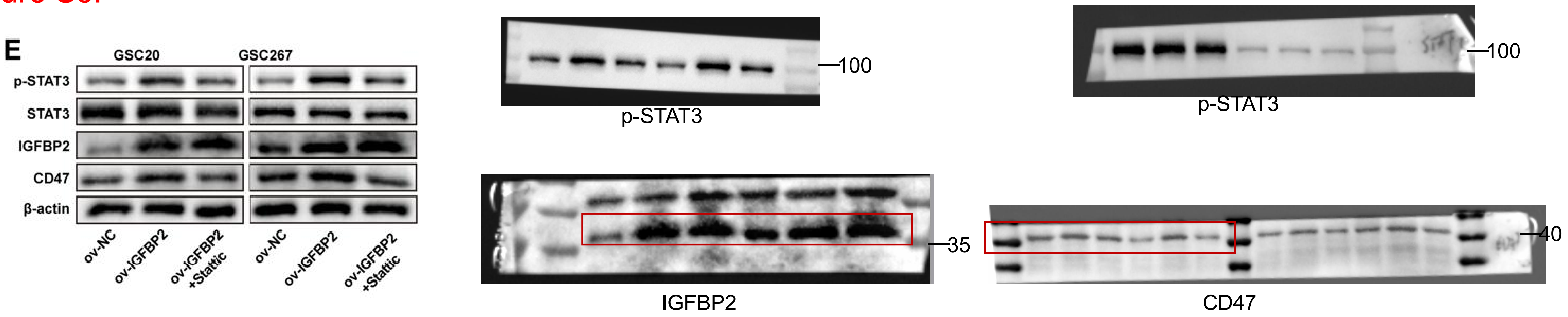

Figure S5J

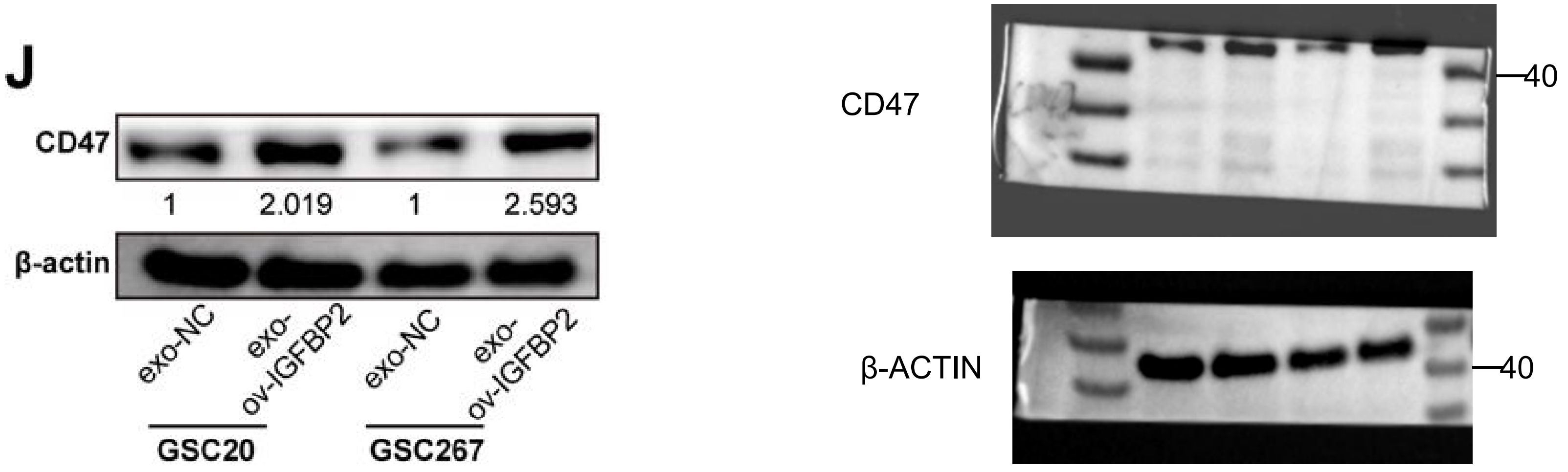

Figure S6C

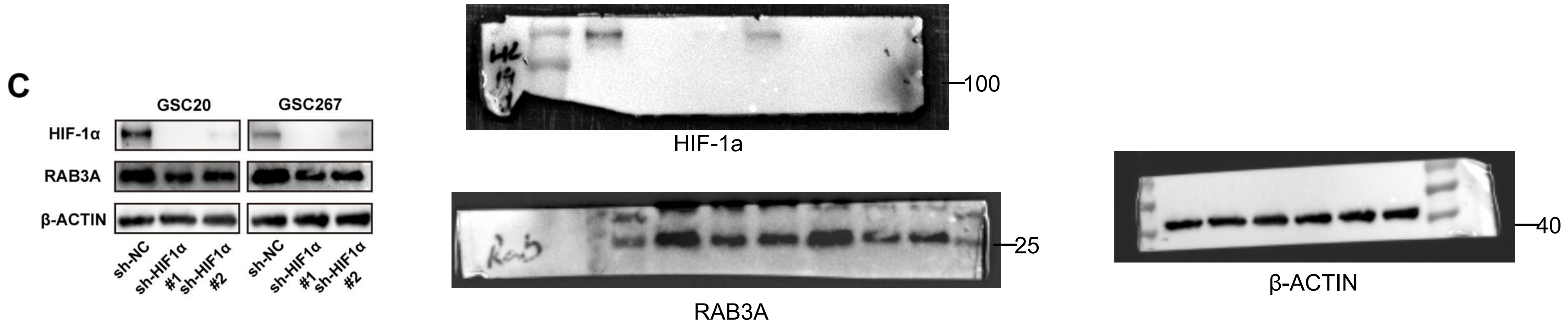

Figure S6D

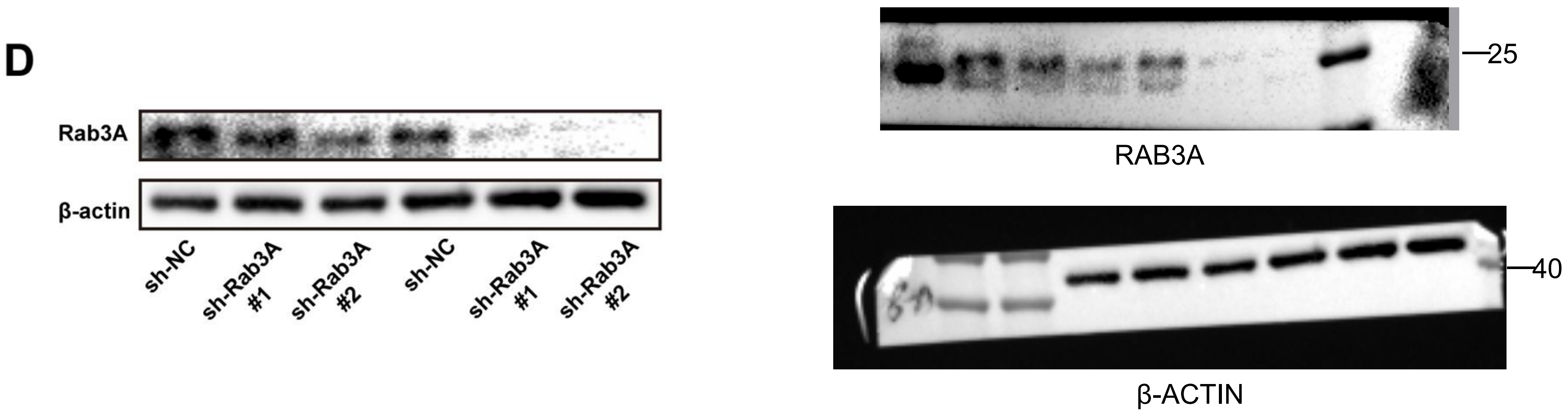

Supplement: Supplementary file 7 — uncropped western blots [file 41419_2026_8430_MOESM7_ESM.pdf]
